# Iodinated Cyanoacrylate‐Based Novel Liquid Embolic Compositions with Inherent Radiopacity for Endovascular Embolization

**DOI:** 10.1002/adhm.202401099

**Published:** 2024-09-05

**Authors:** Seong Ik Jeon, Hyun Jae Park, Hanhee Cho, Saebeom Hur, Young Il Kim, Hwan Jun Jae, Cheol‐Hee Ahn

**Affiliations:** ^1^ Research Institute of Advanced Materials (RIAM) Department of Materials Science and Engineering Seoul National University 1 Gwanak‐ro, Gwanak‐gu Seoul 08826 Republic of Korea; ^2^ Department of Radiology Seoul National University College of Medicine Institute of Radiation Medicine Seoul National University Medical Research Center Clinical Research Institute Seoul National University Hospital 101 Daehak‐ro, Jongno‐gu Seoul 03080 Republic of Korea; ^3^ College of Pharmacy, Graduate School of Pharmaceutical Sciences Ewha Womans University 52 Ewhayeodae‐gil, Seodaemun‐gu Seoul 03760 Republic of Korea

**Keywords:** endovascular embolization, iodinated cyanoacrylate, liquid embolic material, transcatheter interventional delivery, X‐ray contrast agent

## Abstract

Endovascular embolization is a promising therapeutic approach broadening its application area due to its minimal invasiveness and short operation time, wherein lesional blood vessels are occluded with liquid embolic agents under X‐ray imaging guidance. Histoacryl and its composition with Lipiodol are one of the most widely used liquid embolic agents, however, Histoacryl has critical limitations, such as lack of innate X‐ray visibility and strong adhesion to microcatheter. In this study, three different iodinated cyanoacrylates are newly synthesized as alternatives to Histoacryl and employed to develop liquid embolic compositions. Among them, 4‐iodobutyl 2‐cyanoacrylate (IBCA) is most preferable with high iodine content (730 mgI mL^−1^) and fast polymerization. The IBCA‐based embolic compositions containing ethyl oleate and acetic acid showed moderate viscosity and reduced catheter adhesiveness (≈0.80 N), and their polymerization time is freely controllable from 2 to 15 s. In the embolization test with rabbit models, the renal artery is successfully occluded by IBCA‐based embolic compositions without vascular recanalization or nontarget embolization for 4 w. Their embolic effect is further evaluated using swine models, demonstrating the practical applicability in the clinic. In conclusion, IBCA and its compositions are determined to have great potential as novel liquid embolic agents.

## Graphical abstract

Iodinated cyanoacrylate monomers with inherent radiopacity are newly synthesized and employed in developing novel liquid embolic compositions. The iodinated cyanoacrylate‐based liquid embolic compositions exhibit high radiopacity, controllable polymerization rate, and reduced adhesiveness to a microcatheter. Moreover, they show stable embolic effects against not only rabbits but also swine models with human‐comparable vascular morphologies.

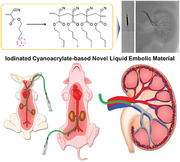

## 1 Introduction

Endovascular embolization is a promising therapeutic method to treat various vascular lesions by deliberately blocking the pathological vessels and ceasing the bloodstream.^[^ ^1^ ^]^ This interventional technique, capable of replacing corresponding surgical procedures, involves catheterizing target vessels under real‐time X‐ray image guidance and delivering embolic agents through a microcatheter. Owing to its minimal invasiveness, endovascular embolization has been increasingly applied to various vascular diseases, such as various internal arterial hemorrhages, arteriovenous malformations (AVM), portal varices, brain aneurysms, and hypervascular tumors.^[^ ^2^ ^]^ With an advancement in embolization techniques, several different embolic agents have been developed including metallic coils, polymeric particles, and liquid compositions.^[^ ^3^ ^]^ Since each embolic agent has its characteristics, it is important to select the most appropriate one according to the type of disease and the condition of the lesion.^[^ ^4^ ^]^

Among the various embolic materials, liquid embolic compositions have attracted considerable attention due to the convenience of transcatheter administration, high efficiency in filling blood vessels, and applicability to vessels with complex morphologies.^[^ ^5^ ^]^ Once injected into target blood vessels through a microcatheter, liquid embolic compositions transform into solid casts after contact with blood. The formed solid casts completely fill vascular lesions and occlude the blood flow regardless of thrombogenesis, unlike other embolic agents, such as metallic coils which have low packing densities and require the formation of blood clots.^[^ ^6^ ^]^ Liquid embolic compositions can be categorized into two distinct types according to their components and solidification mechanism: liquid monomers and polymeric solutions.^[^ ^7^ ^]^ Monomer‐based liquid embolic compositions contain highly reactive monomers that are polymerized immediately upon exposure to moisture or blood components, initiated by them, to form a water‐insoluble polymer mass. Histoacryl (B. Braun) and TruFill (Cordis) are typical reactive monomer‐based liquid embolic agents commercially available. On the other hand, polymer solution‐based embolic agents are composed of biocompatible and water‐insoluble polymers dissolved in organic solvents, which precipitate in blood and solidify into polymeric sponges after the injection.^[^ ^8^ ^]^ Onyx (Medtronic) and PHIL (MicroVention, Inc.) are representative examples of solution‐based embolic agents most extensively utilized in clinical practices. Monomer‐based embolic agents have a rapid polymerization rate, facilitating their application in occluding vasculatures with fast blood flow and high blood pressure, but have a high risk of catheter entrapment with strong adhesiveness.^[^ ^9^ ^]^ In contrast, polymer solution‐based ones exhibit much‐mitigated catheter adhesion due to their prolonged solidification profile but are hard to use in blood vessels with a rapid bloodstream. The organic solvents in the embolic polymer solutions pose complications as well, inducing vasospasm and endothelial cell necrosis and compelling the use of special microcatheters compatible with organic solvents.^[^ ^10^ ^]^

Histoacryl is a monomer‐based liquid embolic agent composed solely of a single substance, n‐Butyl 2‐cyanoacrylate (NBCA).^[^ ^11^ ^]^ Owing to the cyanide and ester groups exerting strong electron‐withdrawing properties within its molecular structure, NBCA undergoes expeditious polymerization even when exposed to weak bases or nucleophiles.^[^ ^12^ ^]^ The highly reactive nature of NBCA enables itself to form a rigid solid immediately, exhibiting a strong and permanent embolic effect when delivered to blood vessels and in contact with anionic substances in the blood. Due to its rapid solidification rate, Histoacryl is mainly used to treat vascular diseases with high blood flow, such as hemorrhages or AVMs.^[^ ^13^ ^]^ However, the utilization of Histoacryl as an embolic agent presents significant drawbacks, including the absence of radiopacity, unpredictable premature polymerization within the microcatheter, and the potential risk of inducing tight adhesion between the catheter tip and the blood vessel.^[^ ^2a^ ^]^ Lipiodol (Guerbet), an iodinated fatty acid ester oil, should inevitably be mixed with Histoacryl to provide X‐ray visibility and regulate the polymerization rate.^[^ ^14^ ^]^ The properties of Histoacryl and Lipiodol compositions (HLs) excessively hinge on the proportion of Lipiodol, with low Lipiodol ratios resulting in a rapid polymerization rate but inadequate radiopacity and strong catheter adhesiveness. Conversely, a high Lipiodol content in HLs ensures their clear X‐ray visibility and reduces the occurrence of catheter entrapment, albeit at the expense of compromising their polymerization rate. Ideally, several properties are essential for embolic agents to improve the safety and effectiveness of embolization procedures, such as sufficient inherent radiopacity, moderate viscosity, adjustable solidification rate, low catheter adhesiveness, and absence of toxic organic solvents, which are difficult to achieve with conventional HLs.^[^ ^3d^ ^]^ In this context, there is a strong requirement for the development of novel cyanoacrylate monomers with intrinsic radiopacity and fast polymerization rate as liquid embolic agents, but not succeeded as of now. This is attributed to the challenge of chemically modifying highly reactive cyanoacrylates while preventing their polymerization and the potential risk of a significant reduction in the polymerization rate of cyanoacrylates after the conjugation of elements with high atomic numbers to impart radiopacity. Therefore, there needs to be close consideration in designing the chemical structure of cyanoacrylate monomers to endow them with both high intrinsic radiopacity and a rapid polymerization rate.

Herein, novel iodinated cyanoacrylates with inherent high radiopacities were employed for the development of liquid embolic compositions overcoming the limitation of Histoacryl. Three iodinated cyanoacrylates with different structures, 2‐iodoethyl 2‐cyanoacrylate (IECA), 4‐iodobutyl 2‐cyanoacrylate (IBCA), and 3‐iodo‐2‐(iodomethyl)−2‐methylpropyl 2‐cyanoacrylate (DIPCA), were synthesized and their basic properties were assessed to identify the most suitable candidate for the preparation of embolic compositions. Subsequently, ethyl oleate and acetic acid were added to iodinated cyanoacrylates to prepare liquid embolic compositions with specifically controlled polymerization time and catheter adhesion strength. After assessing the in vitro biocompatibility of embolic compositions, their visibility through the C‐arm real‐time X‐ray device and their injectability through a microcatheter were determined via the embolization and angiographic analysis of the renal artery in rabbit models. The recipient rabbits were monitored with CT examinations for up to 4 w to assess the long‐term embolic efficacy and detect any possible side effects, including nontarget embolization and hematotoxicity. Finally, the embolic effect of the liquid embolic compositions was further verified in swine models to confirm their applicability in large animals with vasculature comparable to humans, offering valuable insights into their potential effectiveness in a clinically relevant context. These novel iodinated cyanoacrylate‐based liquid embolic compositions would be versatile alternatives to HL compositions clinically used to treat hemorrhages and AVMs.

## 2 Results and Discussion

### 2.1 Characterization of Iodinated Cyanoacrylates

**Scheme 1:**
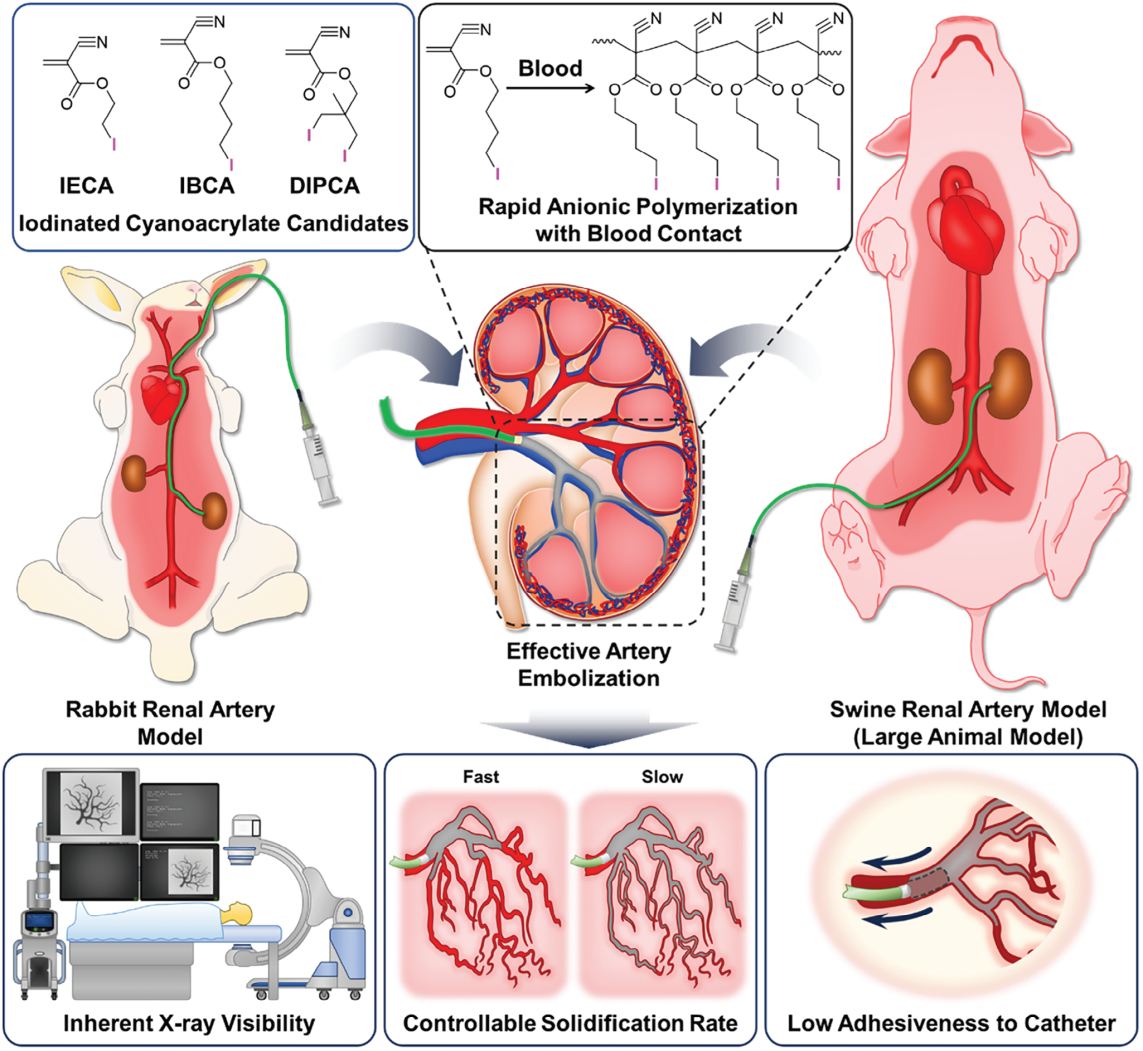
Iodinated cyanoacrylate‐based liquid embolic agents and evaluation of their embolic effect. Three different iodinated cyanoacrylates were newly synthesized and evaluated for their properties as liquid embolic materials. After identifying the most preferable iodinated cyanoacrylate and its embolic composition, its embolic effect was investigated in vivo against rabbit and swine renal artery models. The novel iodinated cyanoacrylate composition developed in this study was expected to be a potent liquid embolic material with high X‐ray visibility during embolization, controllable polymerization time, and low microcatheter adhesiveness.

**Figure 1:**
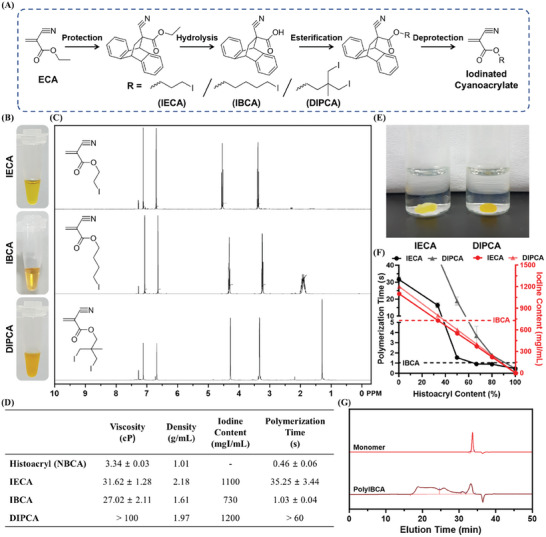
Synthesis and characterization of iodinated cyanoacrylate candidates. A) A synthetic scheme briefly explaining the chemical production of IECA, IBCA, and DIPCA. B) Digital images and C) ^1^H NMR spectra of synthesized IECA, IBCA, and DIPCA. D) A table summarizing the basic properties of synthesized IECA, IBCA, and DIPCA. E) Digital images of IECA and DIPCA dropped into PBS media. F) Blood contact polymerization time and iodine contents of IECA + Histoacryl or DIPCA + Histoacryl mixtures (*n* = 3). G) GPC analysis of polymerized IBCA (Eluent: THF, 1 mL min^−1^).

Iodinated cyanoacrylates with inherent radiopacity were newly developed as liquid embolic compositions and their embolic effect was examined in vivo with rabbit and swine models (**Scheme** **1**). The novel iodinated cyanoacrylate‐based embolic compositions were expected to efficiently address the limitations of conventional HL compositions by providing high radiopacity, controllable polymerization time, and mitigated catheter adhesiveness simultaneously. To obtain the optimal iodinated cyanoacrylate with desirable properties, three candidates with different chemical structures were synthesized through the “protection and deprotection method” utilizing the Diels–Alder cycloaddition between anthracene and cyanoacrylate (**Figure** 1A). Since cyanoacrylate is extremely susceptible to moisture and light irradiation, it is essential to protect its double bond before undergoing further chemical modification. Cyanoacrylates in industrial use, including Histoacryl, are usually synthesized through the depolymerization of polycyanoacrylates, where high temperatures (160–200 °C) are required for the thermal cracking of their main chains.^[^ ^15^ ^]^ Iodide groups in iodinated cyanoacrylate candidates, however, underwent rapid decomposition at temperatures above 160 °C, which hindered their production through the depolymerization method. Therefore, the protection and deprotection method was newly designed to prepare the iodinated cyanoacrylate candidates. The product in each synthetic step was confirmed via proton nuclear magnetic resonance (^1^H NMR) analysis, confirming their successful synthesis with high purity. In the final synthetic steps, pIECA, pIBCA, and pDIPCA were efficiently deprotected with maleic anhydride at a temperature below 150 °C to produce IECA, IBCA, and DIPCA while minimizing the pyrolysis of the iodide group. The resulting iodinated cyanoacrylates were obtained as liquids with light‐ to deep‐yellow colors (Figure 1B). The ^1^H NMR spectra of synthesized IECA, IBCA, and DIPCA are provided in Figure 1C, exhibiting the complete substitution of side chains of cyanoacrylates to 2‐iodoethyl, 4‐iodobutyl, and 3‐iodo‐2‐(iodomethyl)−2‐methylpropyl groups, respectively, without any uncontrolled polymerization of double bonds (7.13–6.66 ppm). It is also worth noting that the purities of all iodinate cyanoacrylate products were precisely controlled to over 99% as impurities included in the monomers may affect their polymerization rates.

After the synthesis of iodinated cyanoacrylate candidates, their basic properties such as densities, iodine contents, viscosities, and polymerization times were measured and described in Figure 1D. IECA, IBCA, and DIPCA exhibit densities of 2.18, 1.61, and 1.97 g mL^−1^, respectively, significantly higher than Histoacryl (1.01 g mL^−1^), attributed to the iodide groups in their molecular structures. IBCA possessed a lower density compared to IECA or DIPCA due to its longer alkyl chain and singular iodine atom content in its molecule. Based on the measured densities and molecular weights of iodinated cyanoacrylates, their theoretical iodine contents were calculated. As expected, DIPCA had the highest iodine content of 1200 mgI mL^−1^, whereas IBCA showed the lowest iodine content (730 mgI mL^−1^). Nevertheless, all three iodinated cyanoacrylates are confirmed to have higher radiopacities than Lipiodol (480 mgI mL^−1^), as the radiopacity of an X‐ray contrast agent is proportional to its iodine content. Subsequently, the dynamic viscosities of iodinated cyanoacrylates were analyzed using a Cannon–Fenske viscometer. The viscosities of IECA and IBCA were 31.62 ± 1.28 and 27.02 ± 2.11 cP, respectively, 8.09–9.47‐fold higher than that of Histoacryl (3.34 ± 0.03 cP) and comparable to that of Lipiodol (26.08 ± 0.94 cP). Moreover, DIPCA showed a viscosity significantly surpassing the confines of the measurement range. The high viscosity of iodinated cyanoacrylates and Lipiodol was attributed to the iodine content within their molecular structures and resulting high densities.^[^ ^16^ ^]^

The polymerization time of the liquid embolic composition is one of the essential factors that should be precisely controlled according to the vascular morphology and blood flow rate for the occlusion of exact target blood vessels. If the polymerization time is too short, the embolic composition may solidify solely at the proximal segment, failing to cast the target blood vessels adequately and eventually leading to the compromised embolic effect.^[^ ^17^ ^]^ Conversely, an excessively delayed polymerization can result in a critical medical accident due to the migration of embolic composition and nontarget embolization.^[^ ^14^, ^18^ ^]^ Iodinated cyanoacrylate candidates were allowed to be polymerized in vitro by contacting the horse blood plasma to quantify their polymerization times (Figure S1, Supporting Information). The polymerization times of IECA and DIPCA were 35.25 ± 3.44 and > 60 s, respectively, over 76.63‐fold longer than that of Histoacryl. The droplets of IECA and DIPCA formed a lens‐like shape rather than spread at the interface with aqueous media, which significantly slowed their polymerization (Figure 1E). It was suggested that the high density and hydrophobicity of IECA and DIPCA limited their spreadability.^[^ ^19^ ^]^ In contrast, the IBCA exhibited much faster polymerization (1.03 ± 0.04 s) than IECA or DIPCA, comparable to Histoacryl (0.46 ± 0.06 s). Although the slow polymerization of IECA of DIPCA could be adjusted by mixing with Histoacryl, over 80% v/v of Histoacryl should be added to shorten their polymerization time in a similar level to pure IBCA, which significantly diminished the iodine content of the mixture (Figure 1F). The iodine contents of IECA and DIPCA compositions mixed with 80% Histoacryl were calculated to be 220 and 240 mgI mL^−1^, respectively. Considering the necessity of incorporating additional compounds to modulate the catheter adhesiveness of the embolic composition, IECA and DIPCA were determined not suitable for embolization due to their exceedingly long polymerization time. Hence, IBCA was finally selected as the most appropriate candidate among the three iodinated cyanoacrylates for the preparation of embolic compositions and subjected to further investigation. The gel permeation chromatography (GPC) peak of polymerized IBCA upon contact with aqueous media is shown in Figure 1G. IBCA was immediately polymerized into polyIBCA with a high molecular weight, which was expected to form a solid embolic cast when injected into blood vessels.

### 2.2 Characterization of IBCA‐Based Embolic Compositions

**Figure 2:**
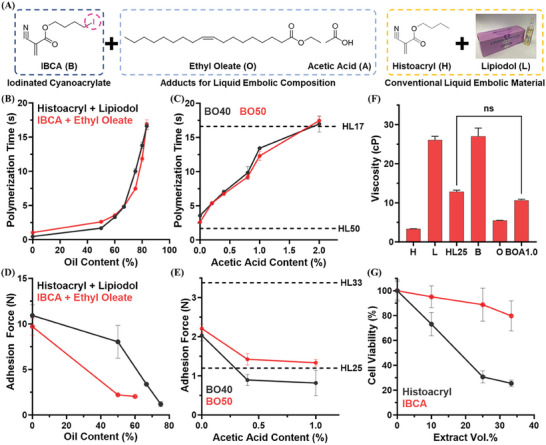
In vitro characterization of IBCA and its embolic compositions. A) Chemical structures of IBCA and adducts composing the IBCA‐based liquid embolic compositions. B) Polymerization time of BOs and HLs according to their oil content after contact with horse blood plasma (*n* = 3). C) Changes in the polymerization time of BO40 and BO50 upon the addition of acetic acid (*n* = 3). D) Catheter adhesion forces of BOs and HLs according to their oil content (*n* = 3). E) Changes in the catheter adhesiveness of BO40 and BO50 upon the addition of acetic acid (*n* = 3). F) Dynamic viscosities of Histoacryl, Lipiodol, IBCA, ethyl oleate, and their compositions (*n* = 5, ns: not significant). G) Cytotoxicity assay of diluted eluents extracted from Histoacryl and IBCA (*n* = 5).

Upon confirming that IBCA has the most favorable iodine content and polymerization behavior among the three iodinated cyanoacrylate candidates, IBCA was blended with various amounts of ethyl oleate and acetic acid to prepare liquid embolic compositions (**Figure** **2**A). Ethyl oleate was added to the embolic compositions as an oil component to control the polymerization time and relieve catheter adhesiveness of IBCA, and acetic acid was to further delay its polymerization time.^[^ ^20^ ^]^ The ethyl oleate content also mitigated the high brittleness of the embolic composition after its polymerization. The prepared liquid embolic composition was stored at 4 °C with a nitrogen purge, which could be maintained over 3 months without any phase separation, polymerization, or decomposition (Figure S2, Supporting Information). The high stability of the embolic composition ensured its shelf‐life extension and quality control. Using the embolic compositions with IBCA and ethyl oleate (BOs) at 17%–50% (based on IBCA content, v/v), the change in its polymerization time depending on the ethyl oleate ratio was investigated first (Figure 2B). The polymerization time of BOs was gradually prolonged as the ethyl oleate content increased, which was freely controllable in a range from 1.03 to 16.88 s. When comparing the polymerization times of BOs to those of HLs depending on the oil content, BOs exhibited a similar polymerization time retardation tendency as HLs even if the polymerization time of pure IBCA was 2.2‐fold longer than that of pure Histoacryl. It was suggested that Lipiodol had a stronger polymerization retardation property than ethyl oleate due to its higher density and hydrophobicity, thereby BOs and HLs showed comparable polymerization times at the same oil content. The high polymerization retardation property of Lipiodol was also demonstrated by measuring the polymerization times of BOs and compositions with IBCA and Lipiodol (Figure S3, Supporting Information).

Although the polymerization time of IBCA could be controlled in a desired range by mixing with ethyl oleate only, it was hard to secure sufficient radiopacity of BOs at high ethyl oleate content since ethyl oleate does not contain any iodine or high atomic number elements. To control the polymerization time of BOs without significant deterioration in their radiopacity, the proportion of IBCA was fixed to 40%–50% (based on IBCA content, v/v; BO40 and BO50) and a small amount of acetic acid was additionally included in BOs (Figure 2C). The polymerization times of BOs could be regulated with the addition of acetic acid as it temporarily inhibited the polymerization of IBCA.^[^ ^17^, ^21^ ^]^ In the clinic, Histoacryl was generally mixed with Lipiodol at percentages ranging from 17% to 50% (based on Histoacryl content, v/v) to control the polymerization time, depending on the size of the target vessel and the blood flow rate.^[^ ^22^ ^]^ Similarly, the polymerization times of BOs could be favorably adjusted to be comparable to those of HLs by mixing acetic acid at concentrations up to 2.0% (based on acetic acid content, v/v), enhancing their practical applicability to various vascular conditions. Since the amount of acetic acid was below 2.0%, the iodine contents of BOs were kept at 290–370 mgI mL^−1^ without any significant reduction, assumed to express sufficient and uniform radiopacity. Moreover, the addition of acetic acid in BOs successfully facilitated the precise control of their polymerization time while constantly maintaining their radiopacity.

The adhesiveness of polymerized embolic compositions to microcatheter is of great importance since the strong catheter adhesiveness may induce the entrapment of the microcatheter after the glue injection and lead to serious medical accidents.^[^ ^9^, ^23^ ^]^ The catheter adhesion force of polymerized BOs in horse blood was measured and compared to that of HLs. The specimen for catheter adhesion force assessment was developed by intentionally entrapping microcatheter tips to BOs or HLs after their polymerization. The adhesion forces were analyzed by a universal tensile machine (Figure S4, Supporting Information). The adhesion force of IBCA without ethyl oleate was measured as 9.70 ± 0.95 N, showing a slightly lower or similar level of catheter adhesiveness compared to pure Histoacryl (10.92 ± 1.17 N) (Figure 2D). The addition of 50% v/v ethyl oleate (BO50) greatly reduced the adhesion strength to 2.21 ± 0.11 N, and the adhesiveness was further mitigated with higher ethyl oleate content (BO40; 2.03 ± 0.14 N). The oily compounds in embolic compositions (i.e., ethyl oleate or Lipiodol) reduce the adhesiveness mainly through two mechanisms; first, the oil acts as a lubricant at the interface between the polymerized cyanoacrylate and microcatheter, and second, it decelerates the polymerization of cyanoacrylate, allowing the solidification to proceed at a site away from the microcatheter.^[^ ^24^ ^]^ Contrary to BOs, there was only a slight reduction in the adhesion strength of Histoacryl after mixing with 50% v/v Lipiodol (HL50; 8.04 ± 1.80 N) since it was immediately polymerized at proximity and tightly entrapped the tip of the microcatheter. As shown in Figure 2E, the catheter adhesiveness of BO further decreased with the addition of acetic acid which delayed the polymerization time of embolic compositions. Notably, BO40s added with acetic acid (BOA) exhibited lower catheter adhesiveness than HLs mixed at ratios of 33% and 25% (based on Histoacryl content, v/v; HL33 and HL25). Considering the polymerization time and catheter adhesiveness of the IBCA compositions, the BO40s added with 0.4% and 1.0% acetic acid (BOA0.4 and BOA1.0) were chosen as the reference composition for the following investigations.

Afterward, the viscosities of liquid embolic compositions were measured to evaluate their injectability through a microcatheter (Figure 2F). Ethyl oleate, an oily compound devoid of any iodine atom in its structure, exhibited a low viscosity of 5.48 ± 0.04 cP, thereby remarkably reducing the viscosity of embolic compounds when mixed with highly viscous IBCA. No significant difference between the viscosities of BOA0.4 and BOA1.0 was found since they had only a minor variance in their compositions, 0.6% acetic acid. Both BOA0.4 and BOA1.0 showed similar viscosity levels compared to HL25, thereby they were expected to be smoothly delivered through the conventional microcatheter during the embolization procedure.

The cytotoxicity of polymerized Histoacryl or IBCA against mouse normal fibroblast (L929) cells was investigated according to the standardized elution test protocol (ISO 10993‐5), wherein polymers did not directly contacted with cells but were extracted with MEM cell culture media at 37 °C for 1 d.^[^ ^25^ ^]^ The extracts were treated on L929 cells after diluting with fresh MEM at ratios of 10%–33% v/v. When L929 cells were incubated with diluted extracts of polymerized Histoacryl or IBCA for 1 d, their viability gradually decreased (Figure 2G). The cytotoxicity of Histoacryl or IBCA extracts was considered to originate from the cyanoacetate and formaldehyde release due to the decomposition of polycyanoacrylates.^[^ ^26^ ^]^ Importantly, the viability of L929 cells treated with diluted IBCA eluents was 1.3–3.2‐fold higher than that of Histoacryl eluent‐treated ones, indicating the significantly alleviated cytotoxicity of IBCA over Histoacryl. It was suggested that polymerized IBCA tends to be decomposed in the extraction media at a slower rate than polymerized Histoacryl due to its higher hydrophobicity, which resulted in the reduced release of cyanoacetate and formaldehyde.^[^ ^27^ ^]^ Given the well‐established biocompatibility of ethyl oleate and acetic acid, BOAs were deemed sufficiently safe for in vivo administration as embolic agents, exhibiting similar or superior biocompatibility compared to HLs.^[^ ^28^ ^]^ Throughout the in vitro evaluation, the embolization applicability and biocompatibility of IBCA and its embolic compositions were comprehensively demonstrated, and the most preferable compositions for in vivo embolization test were also identified.

### 2.3 In Vivo Evaluation of Short‐ and Long‐Term Embolic Effect of IBCA‐Based Embolic Compositions in Rabbit Models

Before the embolization assessment with animal models, the radiopacities of IBCA and its compositions were confirmed in vitro with an artificial soft tissue phantom. The embolic liquids were loaded in 1 mL syringes, placed on the soft tissue phantom, and monitored through the real‐time C‐arm device (Figure S5, Supporting Information). In the real‐time X‐ray image, IBCA showed the highest X‐ray visibility due to its high iodine content (730 mgI mL^−1^), whereas the ethyl oleate which has no iodine in its molecular structure was rarely seen through the device. The visibilities of embolic compositions were found to be completely proportional to their theoretical iodine contents calculated above. The contrast of BOA0.4 or BOA1.0 (290 mgI mL^−1^) was marginally lower than that of HL25 (360 mgI mL^−1^), however, it was still clearly visualized against the soft tissue background.

**Figure 3:**
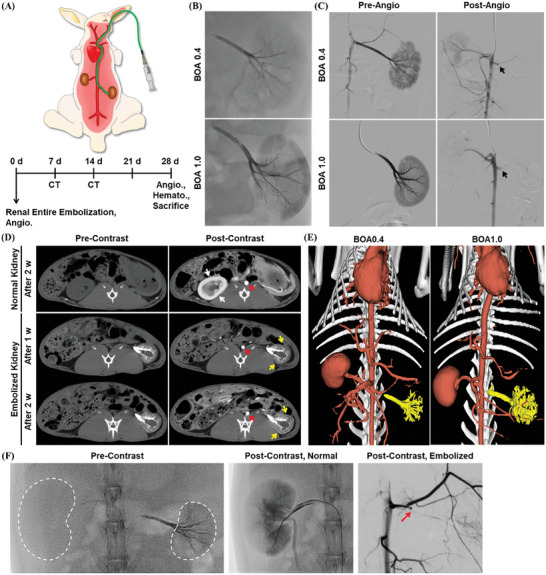
In vivo embolization of rabbit renal artery with BOAs and follow‐up analysis. A) An experimental plan for the rabbit renal artery embolization and follow‐up analysis. B) In vivo real‐time X‐ray images of BOA0.4‐ and BOA1.0‐injected right kidneys of rabbits. C) DSA images of right kidneys before and after the embolization with BOA0.4 and BOA1.0 (black arrow = stagnation of bloodstream). D) 2 w‐follow‐up CTA images of normal and embolized kidneys, before and after the administration of Visipaque320 (red arrow = aorta, white arrow = nonembolized region, yellow arrow = embolized region). E) 3D‐constructed CT images of recipient rabbits showing the embolic casts in embolized kidneys (white = bone, red = blood vessels, yellow = embolic casts). F) Angiography assessment of normal and embolized kidneys 4 w after the embolization (white dashed line = kidneys, red arrow = stagnation of bloodstream).

The short‐ and long‐term embolic effect of BOA0.4 and BOA1.0 was evaluated by injecting them into the renal arteries of New Zealand white rabbits, wherein the general plan for the embolization test and follow‐up analysis was outlined in **Figure** **3**A. The renal artery model was chosen for evaluating the embolic effect of IBCA‐based compositions because renal artery embolization testing is a standardized method for assessing embolic agents with high reproducibility.^[^ ^29^ ^]^ It also provides a robust condition for embolic effect testing due to its high blood supply and flow rate, and it is suitable for long‐term monitoring of embolic effect since one kidney can be embolized, while the other preserves its functionality normally. As a control, HL25, the most commonly utilized embolic composition in the clinical treatment of hemorrhages and AVMs, was also examined, and its embolic efficacy was compared to that of BOA0.4 and BOA1.0. For renal artery embolization, the right renal artery was selected with a microcatheter, and the proper position of its tip was confirmed by pre‐embolization digital subtraction angiography (DSA). Subsequently, 0.50 mL BOA0.4 or BOA1.0 was administered through the microcatheter into the renal artery to achieve complete occlusion of the entire kidney. The embolization tests for each embolic composition were conducted with three animals per experimental group (*n* = 3), with all embolization procedures performed without any notable complications. Both BOA0.4 and BOA1.0 were injected smoothly through the microcatheter, with no instances of premature occlusion of the catheter lumen. The real‐time X‐ray fluoroscopy images during the embolization revealed that both BOA0.4 and BOA1.0 exhibited high radiopacity similar to that of HL25, enabling visualization of the glue cast (Figure 3B; and Figure S6, Supporting Information). The target blood vessels, including peripheral branches, were effectively filled with embolic compositions due to their moderate viscosities and precisely modulated polymerization times. No significant difference in visibility was observed between BOA0.4 and BOA1.0, as their theoretical iodine contents were nearly identical. The microcatheter was freely retractable after the injection of embolic compositions without adhesion or entrapment. DSA was promptly conducted postembolization, showing the total obliteration of the renal artery (Figure 3C; and Videos S1 and S2, Supporting Information). The contrast agents injected for postangiography were unable to pass through the occluded vessels, as indicated by the black arrows. The in vivo radiopacities and embolic outcomes of BOA0.4 and BOA1.0 were found to be not remarkably different from each other and also comparable to those of HL 25.

Recipient rabbits were followed up till 4 w after the procedure with contrast‐enhanced computed tomography (CT) and DSA examination. When the CT angiography (CTA) was conducted before the embolization, both the left and right kidneys were fully highlighted by the injected contrast agents, as the contrast agent flowed into the parenchymal regions of both kidneys (Figure S7, Supporting Information). In contrast, following the embolization, the left kidney occluded with BOA0.4 and BOA1.0 was not enhanced upon contrast agent injection (yellow arrows), whereas the nonembolized right kidney remained enhanced by the contrast (white arrows), indicating that both IBCA‐based embolic compositions effectively impeded blood flow to occluded vessels without any recanalization even 2 w postembolization (Figure 3D; and Figure S8, Supporting Information). The CTA results of rabbit kidneys administered with BOA 0.4 and BOA 1.0 were indistinguishable from those injected with HL25, indicating the sustained embolic effects of both BOA compositions similar to conventional liquid embolic materials (Figure S9, Supporting Information). The CTA data were then reconstructed into 3D images to examine the morphology of embolic casts (Figure 3E). Upon comparing the 3D structures of BOA0.4 and BOA1.0 casts (depicted in yellow) within the kidneys, it was observed that BOA1.0 formed a larger and more widely spread cast. This was attributed to the longer polymerization time of BOA1.0 and its delivery to more distal sites. In the real‐time X‐ray images of both left and right kidneys captured 4 w postembolization, a significant contraction of the embolized right kidney was observed compared to the normal left kidney (Figure 3F; and Figure S10, Videos S3 and S4, Supporting Information). The embolic casts of BOA0.4 and BOA1.0 persistently remained within the left kidney, retaining their visibility in X‐ray images without any reduction in radiopacity. Moreover, repeated DSA analysis conducted 4 w postembolization revealed prolonged obstruction of the bloodstream in the left kidney by BOA0.4 and BOA1.0, while blood flow in the right kidney was kept at normal. Any recanalization of embolized renal arteries due to the fragmentation or degradation of embolic casts was not observed within 4 w, which is highly desirable for the permanent occlusion of hemorrhages or AVMs. Throughout the CTA and DSA analysis, it was determined that both IBCA compositions demonstrated excellent long‐term embolic effects.

### 2.4 Nontarget Embolization and Toxicity Analysis of IBCA‐Based Embolic Compositions

**Figure 4:**
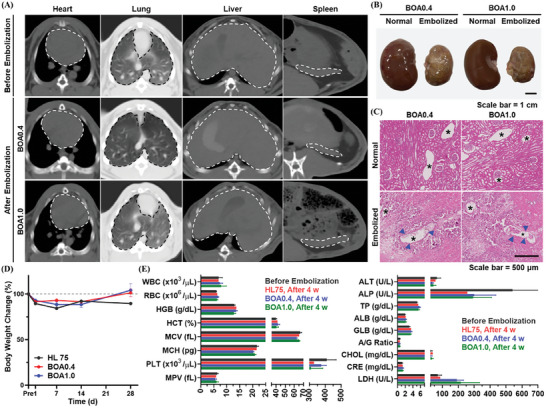
Nontarget embolization and hematotoxicity analysis after the embolization with BOAs. A) Abdominal CT images of rabbits 4 w after embolization with BOA0.4 and BOA1.0, indicating the absence of nontarget embolization (dashed line = organs). B) Digital images of normal and embolized kidneys extracted from recipient rabbits 4 w after embolization. C) Histological analysis of normal and embolized kidneys 4 w after embolization (asterisk = blood vessels, blue arrow = embolic cast). D) Body weight changes of recipient rabbits during 4 w monitoring postembolization (*n* = 3). E) Hematological analysis of recipient rabbits before and 4 w after embolization (*n* = 3).

Evaluating the long‐term embolic effect of IBCA‐based embolic compositions in rabbit models through follow‐up CT and angiography analysis, the investigation focused on the occurrence of nontarget embolization caused by any fragmentation and migration of embolic casts and their hematotoxicity. Owing to the inherent radiopacity of IBCA, it is possible to accurately identify potential fragmentation and translocation of embolic casts through CT imaging, presenting an additional advantage over conventional HL compositions. Abdominal CT images of recipient rabbits taken 2 w after the embolization revealed no signs of undesired migration of radiopaque glue cast (dashed lines), including the heart, lung, liver, and spleen, consistent with the pre‐embolization CT images (**Figure** **4**A; and Figure S11, Supporting Information). All recipient rabbits did not show any noticeable complication of nontarget embolization over a 4 w survival period either, demonstrating the stable durability of polymerized BOA0.4 and BOA1.0 against the blood pressure. Subsequently, the rabbits were sacrificed at 4 w postembolization and their kidneys were harvested for further investigations. In the digital images of both the extracted right and left kidneys, considerable atrophy of the embolized left kidney was evident compared to the untreated one due to the complete infarction and necrosis of renal tissue (Figure 4B). The embolized left kidney also displayed a yellowish color, suggesting a complete blockage of blood supply to the kidney parenchyma by the emboli. When the collected kidneys were sectioned and stained with H&E for histological assay, the embolized kidney lost its original morphology with severe tissue necrosis and polymerized BOA0.4 and BOA1.0 (blue arrows) were observed inside the vascular lumens (asterisks) (Figure 4C). Compared to kidney tissues embolized with HL25, those injected with BOA0.4 and BOA1.0 exhibited a similar tendency toward tissue necrosis (Figure S12, Supporting Information). In contrast, the nonembolized right kidney constantly maintained its tissue integrity without any damage or contamination after the treatment, securing its normal functionality. The in vivo abdominal CT imaging and ex vivo kidney histology indicated that the application of BOA compositions efficiently and selectively occluded the target arteries without affecting other organs or unrelated blood vessels.

The changes in body weights of recipient rabbits were monitored every week for 4 w. BOA0.4‐ and BOA1.0‐injected rabbits experienced moderate weight loss, reducing to ≈88% of their initial weight at 1 and 2 w postembolization (Figure 4D). The temporal weight loss observed in recipient rabbits was attributed to the systemic inflammatory reaction caused by necrosis of the embolized kidney, which was regained to ≈100% by the 4th week. Hematological analysis of recipient rabbits also discovered that all blood components recovered to their normal ranges after 4 w postembolization, inducing no significant immune response or hepatic toxicity (Figure 4E). The outcomes of the in vivo embolization test with rabbit models successfully validated that BOA0.4 and BOA1.0 could promote the site‐specific and prolonged obstruction of target blood vessels with high inertness.

### 2.5 In Vivo Short‐Term Embolic Effect Analysis with Swine Models

**Figure 5:**
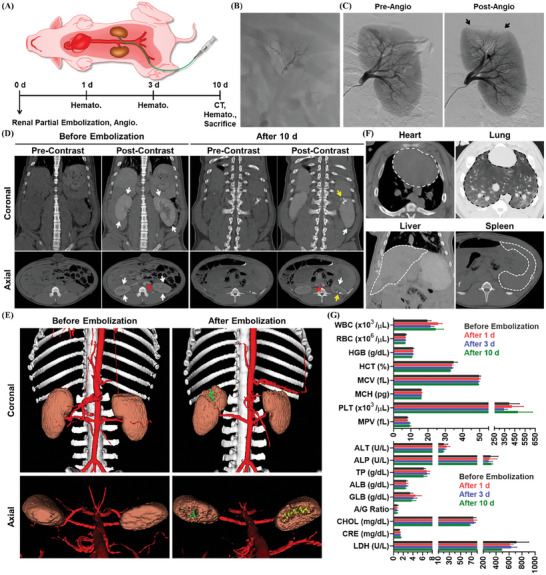
In vivo embolization of swine renal artery with BOAs and follow‐up analysis. A) An experimental plan for the swine renal artery embolization and follow‐up analysis. B) In vivo real‐time X‐ray image of BOA0.4‐injected left kidney. C) DSA images of the left kidney before and after the partial embolization with BOA0.4 (black arrow = embolized region). D) CTA images of normal and embolized kidneys, before and after the administration of Visipaque320 (red arrow = aorta, white arrow = nonembolized region, yellow arrow = embolized region). E) 3D‐constructed CT images of recipient swine showing the embolized regions by HL25 (green) and BOA0.4 (yellow) in kidneys. F) Abdominal CT images of rabbits 10 d after embolization with HL25 and BOA0.4, indicating the absence of nontarget embolization (dashed line = organ). G) 10 d follow‐up hematological analysis of recipient swine (*n* = 3).

To predict the practical applicability of IBCA‐based embolic compositions in actual clinical situations, it is essential to conduct embolization experiments on large animals with vascular structures similar to humans and confirm their embolic effects. Therefore, the embolic effect of BOA0.4 was further examined using swine models, characterized by greater vascular sizes and higher blood pressure compared to rabbits, providing a closer approximation to human vasculature (**Figure** **5**A).^[^ ^28^, ^30^ ^]^ For the embolization of the swine renal artery, BOA0.4 was employed due to its faster polymerization behavior compared to BOA1.0, making it more suitable for occluding larger blood vessels. After the catheterization to the upper renal artery and pre‐DSA analysis, BOA0.4 (1.00 mL) was injected into the blood vessel for the partial embolization of the kidney. As depicted in Figure 5B, the casting of the target blood vessel was clearly visible with the sufficient radiopacity of injected BOA0.4. It was confirmed that BOA0.4 had appropriate viscosity and polymerization rate, firmly embolizing the specific site of intention even in large blood vessels without being washed out by the bloodstream. Figure 5C; and Video S5 (Supporting Information) present the pre‐ and post‐DSA images of embolized vessels, indicating the complete obliteration of blood flow in the BOA0.4‐injected upper renal artery. The in vivo radiopacity and embolic profile of BOA0.4 in large animal models were also comparable to those of HL25, which affirmed the clinical applicability of BOA0.4 (Figure S13, Supporting Information). 10 d after embolization, the embolic cast of BOA0.4 was observed precisely the same as its original state in CT examination, which stagnated the blood flow and prevented the perfusion of the corresponding renal tissue with the contrast agent (yellow arrows) (Figure 5D; and Figure S14, Supporting Information). In contrast, the bloodstream in the nonembolized regions was undisturbed, resulting in contrast enhancement with the smooth transfer of contrast agents in the areas. The embolized upper regions of the kidneys by HL25 (green) and BOA0.4 (yellow) were further visualized through the 3D reconstructed images, wherein the casts of HL25 and BOA0.4 were comparable in size to each other (Figure 5E; and Figure S15, Supporting Information). In abdominal CT images, no unexpected migration of polymerized BOA0.4 fragments into normal organs (dashed lines) was detected for up to 10 d, signifying the stable maintenance of embolic casts (Figure 5F; and Figure S16, Supporting Information). Furthermore, the hematological analysis conducted for 10 d postembolization revealed minimal changes in blood components, attributed not only to the partial embolization of kidneys but also to the biocompatibility of the embolic compositions (Figure 5G). Finally, the comprehensive assessment verified the efficacy of BOA0.4 in blood flow obstruction and embolic durability even in large animals, demonstrating a high potential as an embolic material.

## 3 Conclusion

In this study, novel iodinated cyanoacrylates were proposed as potent liquid embolic agents substituting the conventional embolic compositions of Histoacryl and Lipiodol. Among the three newly synthesized iodinated cyanoacrylate monomers, IBCA was determined to have the most preferable properties, such as moderate density, rapid polymerization, and sufficient iodine content. The IBCA‐based embolic compositions (BOAs) exhibited apparent radiopacity without blending with additional contrast agents, and their polymerization time and catheter adhesiveness could be accurately controlled by adjusting the ethyl oleate and acetic acid contents. BOAs were also proven to have tolerable biocompatibility, displaying moderately lower toxicity against normal cells compared to HLs. In the embolization test with rabbit models, the target renal arteries were successfully occluded by BOAs and the BOA cast in the target artery injected could be precisely visualized in real‐time X‐ray imaging. No fragmentation of the embolic cast or its migration to unintended sites was detected for up to 4 w, and vascular recanalization did not occur either. While embolized kidneys were completely atrophied with severe tissue necrosis, the function of remaining normal kidneys was unaffected, and BOAs did not cause any significant hematotoxicity. Moreover, BOAs could stably block even the renal arteries of swine with large vasculature and high blood pressure as in human, demonstrating their practical applicability in the clinic. In conclusion, BOAs were determined to be a promising liquid embolic agent with high radiopacity, controllable polymerization time, alleviated catheter adhesion, and persistent embolic effect.

## 4 Experimental Section

### Materials

Ethyl 2‐cyanoacrylate (ECA), anthracene (99%), oxalyl chloride (≥ 99%), *N,N*‐dimethylformamide (DMF; anhydrous, 99.8%), triethylamine (TEA; ≥ 99%), 1,4‐butanediol (≥ 99%), 1,1,1‐tris(hydroxymethyl)ethane (≥ 98%), iodine (≥ 99.8%), triphenylphosphine (99%), imidazole (99%), *p*‐xylene (anhydrous, ≥ 99%), maleic anhydride (99%), hydroquinone (≥ 99%), phosphorus pentoxide (99%), ethyl oleate (98%), and acetic acid (≥ 99.7%) were purchased from Sigma‐Aldrich (St. Louis, MO). 2‐Iodoethanol (≥ 98%) was obtained from Tokyo Chemical Industry (TCI; Tokyo, Japan). Benzene (99.5%), ethanol (99.9%), potassium hydroxide (93%), hydrochloric acid (HCl; 35%), diethyl ether (99.0%), tetrahydrofuran (THF; 99.5%), and chloroform (99.5%) were commercially available from Daejung (Siheung, Korea). Histoacryl and Lipiodol were obtained from B. Braun (Melsungen, Germany) and Guerbet (Aulnay‐Sous‐Bois, France), respectively. Horse blood (defibrinated) was purchased from KisanBio (Seoul, Korea). TEA, benzene, and chloroform were dried with calcium hydride, and diethyl ether and THF were distilled over sodium benzophenone ketyl before use. 1,1,1‐Tris(hydroxymethyl)ethane was recrystallized in benzene. Other chemicals were used without further purification.

### Animal Models

New Zealand white rabbits (3.0–3.5 kg) and domestic swine (30 kg) were used in this investigation. In advance of the embolization, all rabbits were anesthetized with an intramuscular injection of tiletamine‐zolazepam (Zoletil 50; Virbac, France; 15 mg kg^−1^). In the case of swine models, Zoletil 50 (5 mg kg^−1^) was intramuscularly administrated for anesthesia. An in vivo examination was conducted at Seoul National University Hospital. All research was authorized by the Institutional Animal Care and Use Committee in Seoul National University Hospital (SNUH‐IACUC), and animals were kept in a facility accredited by AAALAC International (#0 01169) in conformity with the Guide for the Care and Use of Laboratory Animals (IACUC No. 22‐0078‐S1A1(2)).^[^ ^31^ ^]^

### Instruments

^1^H NMR analysis was carried out with Bruker Advance 300 MHz spectrometer (Bruker, Germany) using CDCl_3_ or DMSO‐d6 as a solvent. The molecular weight of polymerized cyanoacrylate was measured via GPC (LC‐20AD; Shimadzu, Japan) using THF as the eluent (elution rate: 1.0 mL min^−1^, elution time: 50 min). A universal tensile machine (Quasar 5; Galdabini, Italy) was used to measure the adhesion strength of embolic agent samples against the microcatheter through a simple tensile method. In vitro cytotoxicity of polymerized cyanoacrylates was assessed using a microplate reader (Infinite M Nano, Tecan., Männedorf, Switzerland). In vitro and in vivo radiopacity of samples was investigated with a real‐time C‐arm X‐ray device (Dyna CT; Siemens, Germany).

### Synthesis of Iodinated Cyanoacrylates

Iodinated cyanoacrylates, including 2‐iodoethyl 2‐cyanoacrylate (IECA), 4‐iodobutyl 2‐cyanoacrylate (IBCA), and 3‐iodo‐2‐(iodomethyl)−2‐methylpropyl 2‐cyanoacrylate (DIPCA), were synthesized through the protection‐deprotection method using Diels–Alder cycloaddition reaction. Briefly, the highly reactive double bond of ECA was protected with anthracene, its ethyl acrylate was successively hydrolyzed under a basic condition, iodoalkyl groups were introduced via esterification, and the protective anthracene was finally removed to produce iodinated cyanoacrylates. All synthetic steps were confirmed via ^1^H NMR analysis, and synthesized IBCA was sampled and polymerized upon water contact for 5 min to analyze via GPC. The synthetic procedure for each iodinated cyanoacrylate was precisely addressed in the Supporting Information.

### Preparation of Cyanoacrylate Compositions

The embolic compositions containing IBCA and ethyl oleate were prepared by mixing them at various volume ratios ranging from 20% to 50% (based on IBCA content, v/v). Anhydrous acetic acid with high purity (≥ 99.7%) was also added to the mixtures at 0.2%– 2.0% v/v for further control of their polymerization time. Each component was placed into a nitrogen‐blown 4 mL glass vial at a fixed ratio and homogenously blended by moderate agitation. The mixed embolic compositions were tightly sealed with Parafilm after a nitrogen purge and stored at 4 °C. The compositions of Histoacryl and Lipiodol (20%–50%, based on Histoacryl content, v/v) were also prepared via the same procedure as controls.

### Polymerization Time Measurement

The polymerization times of iodinated cyanoacrylates and their compositions with ethyl oleate and acetic acid were measured using horse blood plasma as described elsewhere.^[^ ^32^ ^]^ Briefly, a slide glass (25.00 mm × 75.00 mm) was washed with ethanol, dried, and placed on paper imprinted with newsprint‐sized lettering. After dropping 50 µL of blood plasma onto the slide glass, a droplet (10 µL) of the embolic composition sample was allowed to contact the plasma. The time until the letters on paper became invisible through the glass and blood plasma with the complete polymerization of cyanoacrylate was measured. This procedure was repeated three times for each sample and the average polymerization time was calculated.

### Determination of Catheter Adhesion Strength

The adhesiveness between polymerized cyanoacrylate compositions in blood and the tip of the microcatheter was measured in vitro. The whole blood of the horse (0.25 mL) was introduced in a 0.50 mL polypropylene well (0.25 cm in diameter × 3.00 cm in height). 2.00 cm of a microcatheter tip (Progreat, 2.0 Fr, Terumo, Japan) was dipped in the horse blood, and embolic compositions (0.20 mL) were injected into the well through the microcatheter. The injected cyanoacrylates or mixed compositions were allowed to polymerize completely with the blood contact for 30 min, and the microcatheter tip was immobilized in the mixture during the polymerization to induce its deliberate entrapment. The adhesion force was measured through a simple tensile mode at a constant tensile speed of 1.00 mm min^−1^ and the test was performed three times with each sample to obtain the average value.

### Viscosity Analysis

The dynamic viscosities of iodinated cyanoacrylates or their compositions were measured with a Cannon–Fenske viscometer. To prevent the polymerization of cyanoacrylates during the analysis, the viscometer was treated with 0.1 N HCl, washed 3 times with acetone, and dried under vacuum before use. The viscometer was then submerged in a 37 °C thermostatic bath and 4.00 mL embolic liquids were drawn into it. After measuring the time required for embolic liquids to pass through the capillary, the viscosity was calculated according to the following equation

(1) μ=k·ρ·t

where *µ*, *k*, *ρ*, and *t* are the dynamic viscosity (cP), viscometer constant, the density of embolic liquids (g mL^−1^), and time (*s*), respectively. The test was carried out five times for each sample and their average was obtained.

### In Vitro Cytotoxicity Test

The cytotoxicity of cyanoacrylate was investigated against L929 cells according to ISO10993‐5, the minimum essential media (MEM) elution method without any direct contact between polymerized cyanoacrylates and the cells.^[^ ^33^ ^]^ Before the cytotoxicity assay, L929 cells were seeded and cultured in 96‐well plates (0.5 × 10^4^ cells per well) for 1 d. Cyanoacrylate monomers (50 µL) were separately polymerized in MEM (1.00 mL) and incubated at 37 °C for 1 d. The mixture was centrifuged at a rate of 500 xg for 5 min to obtain extracts of polymerized cyanoacrylates, and the extracts were successively diluted with fresh MEM at ratios of 10%–33% v/v. L929 cells cultured in 96‐well plates were incubated with the diluted extract media for 1 d, and cell viability was quantified using a cell counting kit‐8 (CCK‐8) assay.

### In Vivo Embolization Test with Rabbit Models

In vivo embolization test was performed on the renal arteries of New Zealand white rabbits. Three different embolic compositions were prepared before the procedure, BOA0.4 (IBCA/ethyl oleate/acetic acid, containing 60% v/v oil and 0.4% v/v acetic acid), BOA1.0 (IBCA/ethyl oleate/acetic acid, containing 60% v/v oil and 1.0% v/v acetic acid), and HL25 (Histoacryl/Lipiodol, containing 75% v/v oil). The embolization tests with each embolic composition were repeated three times for all groups of recipient rabbits (*n* = 3). Under anesthesia, a 21 G angiocatheter was inserted into the right auricular artery of the rabbit and a 2.0 Fr Progreat microcatheter was subsequently introduced through the sheath. The catheter tip was specifically placed in the right renal artery using a guidewire, and the preoperative angiography was performed with an injection of a nonionic X‐ray contrast agent (iodixanol, Visipaque 320; GE Healthcare, IL). After angiographic identification of the renal artery, the embolic composition (0.50 mL) was administered immediately after the catheter was flushed with 5% dextrose solution. During the injection, the in vivo radiopacity of the embolic composition was observed using a real‐time C‐arm X‐ray device. Digital subtraction angiography (DSA) of the embolized artery was carried out, thereafter, to evaluate the embolic effect of each sample. The recipient rabbits underwent whole‐body CT imaging with intravenous contrast injection at 1 and 2 w after embolization procedures, and the blood flow obstruction persistency was reassessed at 4 w postembolization through DSA with a C‐arm X‐ray device. Afterward, all rabbits were euthanized at 4 w after the embolization, blood samples were obtained for hematological assay, and their kidneys were removed to further proceed with the histological and optical investigation. The normal and embolized kidneys harvested from recipient rabbits were immobilized in 4% paraformaldehyde and their sections were stained with hematoxylin and eosin (H&E) for the microscopic histology analysis.

### In Vivo Embolization Test with Swine Models

After domestic swine (*n* = 3) was anesthetized, the left renal artery was selected using 5F angiocatheter through the transfemoral approach. Upon conducting DSA of renal arteries, BOA0.4 and HL25 (1.00 mL, respectively) were separately delivered to the upper renal arteries of the left and right kidneys through the microcatheter, respectively, right after the inner space of the catheter was flushed out with a 5% dextrose solution. The in vivo radiopacity of the embolic composition was monitored during the administration via a real‐time C‐arm X‐ray device, and DSA was carried out again to confirm the embolic effect. The blood samples were collected from the recipient swine at 1, 3, and 10 d after the embolization, and whole‐body CT imaging was conducted at 10 d postembolization.

### Statistical Analysis

No preprocessing of data was performed for any statistical analyses. All statistical data were expressed as means ± standard error of the mean (SEM) based on at least three repetitions (*n* ≥ 3). Statistical differences were determined using a Student's *t*‐test or the one‐way analysis of variance (ANOVA), with a *p*‐value of less than 0.05 was considered statistically significant. GraphPad Prism 8 software was utilized for graph drawing and statistical analysis. not significant (ns) *p* > 0.05, *p < 0.05, **p < 0.01, ***p < 0.001, ****p < 0.0001.

## Conflict of Interest

The authors declare no conflict of interest.

## Supporting information

Supporting Information

Supplemental Video 1

Supplemental Video 2

Supplemental Video 3

Supplemental Video 4

Supplemental Video 5

## Data Availability

The data that support the findings of this study are available from the corresponding author upon reasonable request.
