# Supplementary material for: Iodinated Cyanoacrylate‐Based Novel Liquid Embolic Compositions with Inherent Radiopacity for Endovascular Embolization
Source: Adv Healthc Mater. 2024 Sep 5;13(29):2401099. doi: 10.1002/adhm.202401099 (PMC11582500; doi:10.1002/adhm.202401099)
Supplement: Supplementary file 1 — Supporting Information [file ADHM-13-0-s006.docx]

*Supporting Information for Advanced Healthcare Materials*

**Iodinated Cyanoacrylate-based Novel Liquid Embolic Compositions with Inherent Radiopacity for Endovascular Embolization**

Seong Ik Jeon, Hyun Jae Park, Hanhee Cho, Saebeom Hur, Young Il Kim, Hwan Jun Jae^*^, and Cheol-Hee Ahn^*^

S. I. Jeon, H. J. Park, H. Cho, C.-H. Ahn

Research Institute of Advanced Materials (RIAM), Department of Materials Science and Engineering

Seoul National University

1 Gwanak-ro, Gwanak-gu, Seoul 08826, Republic of Korea

E-mail: chahn@snu.ac.kr

S. Hur, Y. I. Kim, H. J. Jae

Department of Radiology, Seoul National University College of Medicine, Institute of Radiation Medicine, Seoul National University Medical Research Center, Clinical Research Institute

Seoul National University Hospital

101 Daehak-ro, Jongno-gu, Seoul 03080, Republic of Korea

E-mail: jaemdphd@gmail.com

^*^Corresponding authors.

**1. Supplementary explanation for iodinated cyanoacrylate synthesis**

1.1. Protected ECA (pECA) synthesis

Anthracene (45.00 g, 252.49 mmol) was placed into a 500 mL two-neck round-bottom flask and 200 mL anhydrous benzene was added to make a slurry. After stirring and bubbling with nitrogen gas for 2 h, a reflux condenser was attached to the system and the mixture was heated to reflux condition to make a clear solution. ECA (9.94 mL, 84.16 mmol) was then added to the solution and the reaction was carried out for 1 d under a nitrogen atmosphere. The resulting mixture was cooled down to room temperature, concentrated, and precipitated in a 10-fold excess volume of diethyl ether to remove the unreacted anthracene (yield: 92 %). ^1^H NMR (CDCl_3_, δ, ppm) = 7.54-7.12 (m, 8 H), 4.92 (s, 1 H), 4.45 (s, 1 H), 4.26-4.10 (m, 2 H), 2.87-2.20 (m, 2 H), 1.32-1.28 (t, *J* = 6.9 Hz, 3 H).

1.2. Hydrolysis of ethyl ester in pECA

pECA (23.00 g, 75.82 mmol) was dissolved in 100 mL ethanol and a 5 M potassium hydroxide aqueous solution (20.00 mL) was added. The mixture was allowed to react at 60 ℃ for 2 h. After the reaction, the ethanol was evaporated and 1 N HCl solution was added to neutralize the potassium hydroxide. The crude was extracted with diethyl ether/H_2_O 3 times and the organic layer was dried over magnesium sulfate, concentrated, and precipitated in chloroform. Protected 2-cyanoacrylic acid (pCAcid), the resulting product, was dried in a 25 ℃ vacuum oven for 1 d to obtain a white powder. (yield: 96%). ^1^H NMR (DMSO-d6, δ, ppm) = 7.54-7.08 (m, 8 H), 4.98 (s, 1 H), 4.56 (s, 1 H), 2.63-2.05 (m, 2 H).

1.3. Esterification of pCAcid with 2-iodoethanol

pCAcid (20.00 g, 72.65 mmol) and 200 mL anhydrous diethyl ether were placed into a 500 mL two-neck round-bottom flask equipped with a nitrogen purge and cooled down to 0 ℃. A catalytic amount of anhydrous DMF (0.50 mL) and oxalyl chloride (7.48 mL, 87.17 mmol) were then successively added and the mixture was allowed to react for 3 h. When the mixture was turned into a yellowish-clear solution, the solvent and HCl, a side product, were removed under reduced pressure. The resulting product was immediately dissolved in 200 mL anhydrous diethyl ether again and 2-iodoethanol (5.95 mL, 76.28 mmol) was added to the solution. During the esterification reaction, the mixture was chilled with an ice bath, and triethylamine (10.63 mL, 76.28 mmol) was slowly added using a syringe pump (1.00 mL/min) to remove the side product, HCl. The resulting mixture was filtrated, concentrated, and purified by column chromatography on silica gel to obtain protected 2-iodoethyl 2-cyanoacrylate (pIECA). (yield: 94%). ^1^H NMR (CDCl_3_, δ, ppm) = 7.53-7.12 (m, 8 H), 4.95 (s, 1 H), 4.47-4.45 (t, *J* = 2.4 Hz, 1 H), 4.38-4.32 (m, 2 H), 3.28-3.23 (m, 2 H), 2.86-2.22 (m, 2 H).

1.4. Deprotection of pIECA

pIECA (29.00 g, 67.56 mmol) was put into a 500 mL two-neck round-bottom flask and dissolved in 250 mL anhydrous *p*-xylene. After adding hydroquinone (0.37 g, 3.38 mmol) and phosphorus pentoxide (1.92 g, 6.76 mmol), the mixture was stirred and bubbled with nitrogen gas for 2 h. Attaching the reflux condenser, maleic anhydride (13.25 g, 135.12 mmol) was added and the deprotection of pIECA was carried out for 1 d under reflux condition. The resulting mixture was concentrated, precipitated in anhydrous diethyl ether, and filtrated to collect a clear solution. After evaporating the solvent, the crude product was distilled under reduced pressure to obtain pure 2-iodoethyl 2-cyanoacrylate (IECA). (yield: 74%) ^1^H NMR (CDCl_3_, δ, ppm) = 7.13 (s, 1 H), 6.69 (s, 1 H), 4.56-4.51 (t, *J* = 7.1 Hz, 2 H), 3.40-3.35 (t, *J* = 6.9 Hz, 2 H).

1.5. Esterification of pCAcid with 1,4-butanediol

pCAcid (20.00 g, 72.65 mmol) was placed into a 500 mL two-neck round-bottom flask equipped with a nitrogen purge, dissolved with 200 mL anhydrous diethyl ether, and the solution was cooled down to 0 ℃. Anhydrous DMF (0.50 mL) and oxalyl chloride (7.48 mL, 87.17 mmol) were added to the solution and the mixture was allowed to react for 3 h. After the reaction, the solvent and acidic side product were evaporated to obtain a light yellowish solid. The resultant was dissolved with 50 mL anhydrous THF and transferred to another 500 mL two-neck round-bottom flask containing the solution of 1,4-butanediol (32.09 mL, 363.23 mmol) in 300 mL THF. While adding triethylamine (10.63 mL, 76.28 mmol) in a dropwise manner (1.00 mL/min), the mixture was allowed to react at 0 ℃ for 3 h under a nitrogen atmosphere. The resulting mixture was filtrated, concentrated, and extracted with diethyl ether/H_2_O 3 times. The organic layer was dried with magnesium sulfate and the solvent was evaporated to obtain protected 4-hydroxybutyl 2-cyanoacrylate (pBCA-OH). (yield: 81%). ^1^H NMR (CDCl_3_, δ, ppm) = 7.53-7.14 (m, 8 H), 4.89 (s, 1 H), 4.47-4.45 (t, *J* = 2.4 Hz, 1 H), 4.18-4.12 (m, 2 H), 3.73-3.69 (t, *J* = 6.0 Hz, 2 H), 2.85-2.21 (m, 2 H), 1.79-1.65 (m, 4 H).

1.6. Iodination of pBCA-OH

pBCA-OH (20.00 g, 57.57 mmol) was placed into a 500 mL two-neck round-bottom flask and dissolved with 250 mL anhydrous chloroform. Triphenylphosphine (22.65 g, 86.35 mmol), imidazole (5.88 g, 86.35 mmol), and iodine (21.92 g, 86.35 mmol) were successively added to the solution and the reaction was allowed to proceed at 40 ℃ for 1 d under nitrogen atmosphere. After the reaction, the mixture was concentrated and precipitated in diethyl ether to remove imidazolium iodide salt. The filtrated clear solution was collected and purified by column chromatography on silica gel to obtain protected 4-iodobutyl 2-cyanoacrylate (pIBCA). (yield: 93%). ^1^H NMR (CDCl_3_, δ, ppm) = 7.54-7.15 (m, 8 H), 4.90 (s, 1 H), 4.47-4.46 (t, *J* = 2.7 Hz, 1 H), 4.18-4.11 (t, *J* = 6.0 Hz, 2 H), 3.26-3.21 (m, 2 H), 2.85-2.22 (m, 2 H), 1.90-1.78 (m, 4 H).

1.7. Deprotection of pIBCA

pIBCA (24 g, 52.48 mmol) was placed into a 500 mL two-neck round-bottom flask and dissolved in 200 mL anhydrous *p*-xylene. Adding hydroquinone (0.29 g, 2.62 mmol) and phosphorus pentoxide (1.49 g, 5.25 mmol), the mixture was stirred and bubbled with nitrogen gas for 2 h. After attaching a reflux condenser, maleic anhydride (10.29 g, 104.96 mmol) was added and the mixture was heated to reflux and allowed to react for 1 d under a nitrogen atmosphere. The resultant was then concentrated, and precipitated in anhydrous diethyl ether, and the clear solution was collected. After evaporating the solvent, the crude mixture was distilled under a vacuum to obtain 4-iodobutyl 2-cyanoacrylate (IBCA). (yield: 72%) ^1^H NMR (CDCl_3_, δ, ppm) = 7.08 (s, 1 H), 6.66 (s, 1 H), 4.35-4.31 (t, *J* = 6.3 Hz, 2 H), 3.27-3.23 (t, *J* = 6.6 Hz, 2 H), 2.01-1.89 (m, 4 H).

1.8. Esterification of pCAcid with 1,1,1-tris(hydroxymethyl)ethane

pCAcid (10.00 g, 36.33 mmol) was placed into a 500 mL two-neck round-bottom flask equipped with a nitrogen purge, dissolved with 200 mL anhydrous diethyl ether, and the solution was cooled down to 0 ℃. Anhydrous DMF (0.30 mL) and oxalyl chloride (3.74 mL, 43.59 mmol) were added to the solution and the mixture was allowed to react for 3 h. After the reaction, the solvent and acidic side product were evaporated to obtain a light yellowish solid. The resultant was dissolved with 50 mL anhydrous THF and transferred to another 500 mL two-neck round-bottom flask containing the solution of 1,1,1-tris(hydroxymethyl)ethane (43.65 g, 363.23 mmol) in 300 mL THF. While adding triethylamine (5.32 mL, 38.14 mmol) in a dropwise manner (1.00 mL/min), the mixture was allowed to react at 0 ℃ for 3 h under a nitrogen atmosphere. The resulting mixture was filtrated, concentrated, and extracted with chloroform/H_2_O 3 times. The organic layer was dried with magnesium sulfate, concentrated, and separated *via* column chromatography on silica gel to obtain protected 3-hydroxy-2-(hydroxymethyl)-2-methylpropyl 2-cyanoacrylate (pPCA-Diol). (yield: 71%). ^1^H NMR (CDCl_3_, δ, ppm) = 7.54-7.15 (m, 8 H), 4.89 (s, 1 H), 4.48-4.46 (t, *J* = 2.7 Hz, 1 H), 4.29-4.07 (m, 2 H), 3.62-3.54 (m, 4 H), 2.84-2.23 (m, 2 H), 0.90 (s, 3H).

1.9. Iodination of pPCA-Diol

pPCA-Diol (10.00 g, 26.49 mmol) was placed into a 500 mL two-neck round-bottom flask and dissolved with 250 mL anhydrous chloroform. Triphenylphosphine (20.85 g, 79.48 mmol), imidazole (5.41 g, 79.48 mmol), and iodine (20.17 g, 79.48 mmol) were successively added to the solution and the reaction was allowed to proceed at 40 ℃ for 1 d under nitrogen atmosphere. After the reaction, the mixture was concentrated and precipitated in diethyl ether to remove imidazolium iodide salt. The filtrated clear solution was concentrated and separated by column chromatography on silica gel to obtain protected 3-iodo-2-(iodomethyl)-2-methylpropyl 2-cyanoacrylate (pDIPCA). (yield: 94%). ^1^H NMR (CDCl_3_, δ, ppm) = 7.56-7.17 (m, 8 H), 4.85 (s, 1 H), 4.49-4.47 (t, *J* = 2.4 Hz, 1 H), 4.22-4.3.97 (m, 2 H), 3.32-3.28 (m, 2 H), 2.83-2.24 (m, 2 H), 1.25 (s, 3 H).

1.10. Deprotection of pDIPCA

pDIPCA (10 g, 16.74 mmol) was placed into a 500 mL two-neck round-bottom flask and dissolved in 200 mL anhydrous *p*-xylene. Adding hydroquinone (0.09 g, 0.84 mmol) and phosphorus pentoxide (0.48 g, 1.67 mmol), the mixture was stirred and bubbled with nitrogen gas for 2 h. After attaching a reflux condenser, maleic anhydride (3.28 g, 33.49 mmol) was added and the mixture was heated to reflux and allowed to react for 1 d under a nitrogen atmosphere. The resultant was then concentrated, and precipitated in anhydrous diethyl ether, and the clear solution was collected. After evaporating the solvent, the crude mixture was washed three times with n-hexane and dried to obtain 3-iodo-2-(iodomethyl)-2-methylpropyl 2-cyanoacrylate (DIPCA). (yield: 90%) ^1^H NMR (CDCl_3_, δ, ppm) = 7.13 (s, 1 H), 6.69 (s, 1 H), 4.29 (s, 2 H), 3.38-3.31 (m, 2 H), 1.30 (s, 3 H).

**2. Supplementary scheme and figures**

**Figure S1.**

**
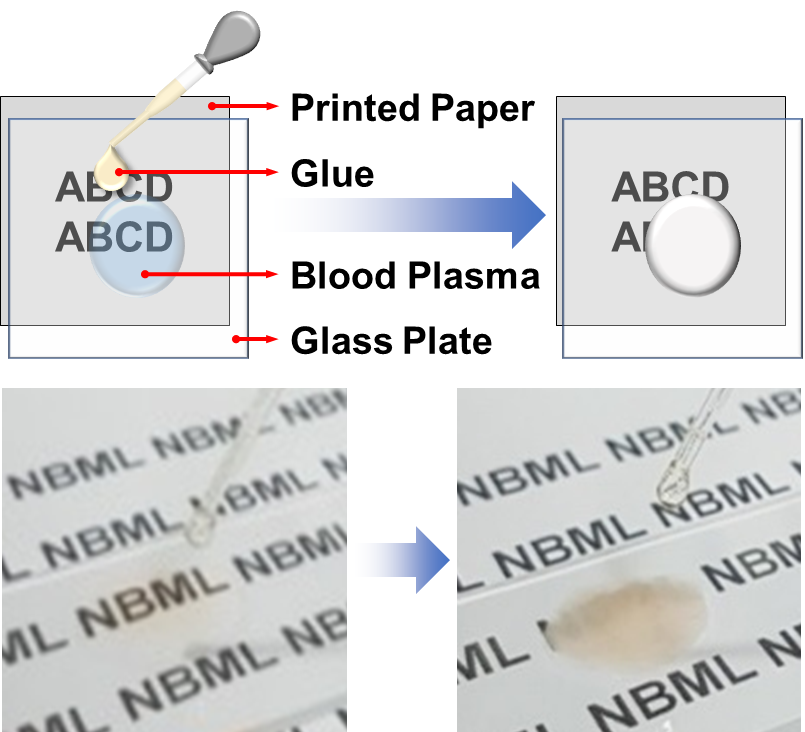
**

**Figure S1.** Schematic illustration depicting the method to measure the polymerization time of cyanoacrylates and their embolic compositions using horse blood plasma.

**Figure S2.**

**
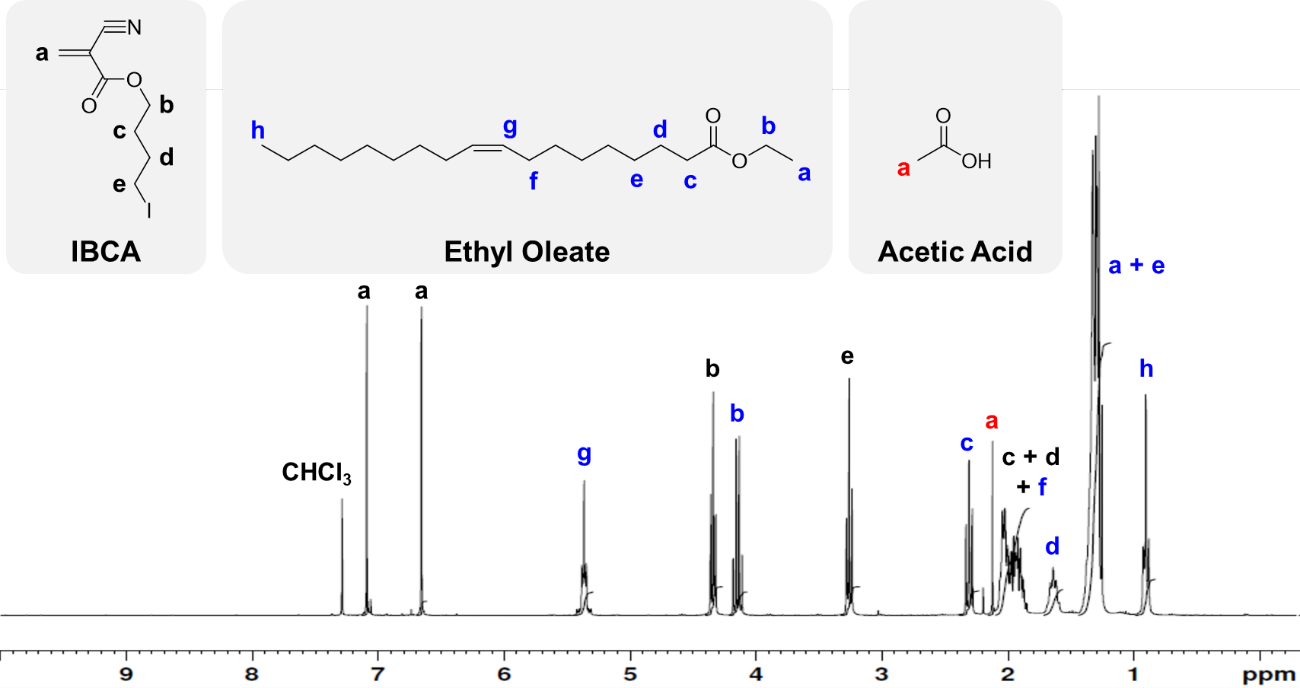
**

**Figure S2.** ^1^H NMR spectra of BOA0.4 after being stored at 4 ℃ for 3 months.

**Figure S3.**

**Figure S3.** Changes in the polymerization time of IBCA-based compositions as a function of the proportion of Lipiodol and ethyl oleate (n = 3).

**Figure S4.**

**
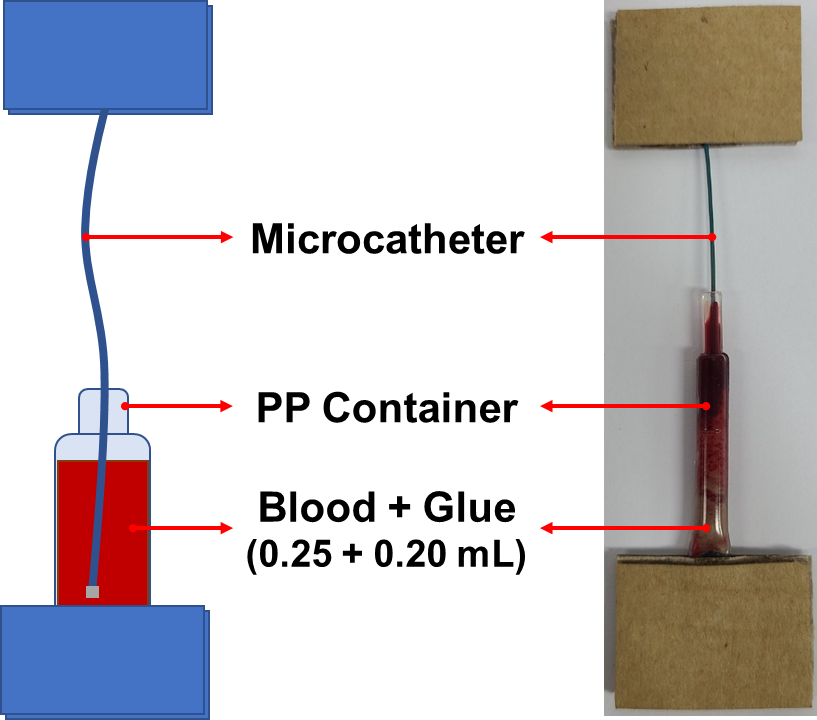
**

**Figure S4.** An illustration and a digital image of a specimen to measure the microcatheter adhesion strength of embolic compositions.

**Figure S5.**

**
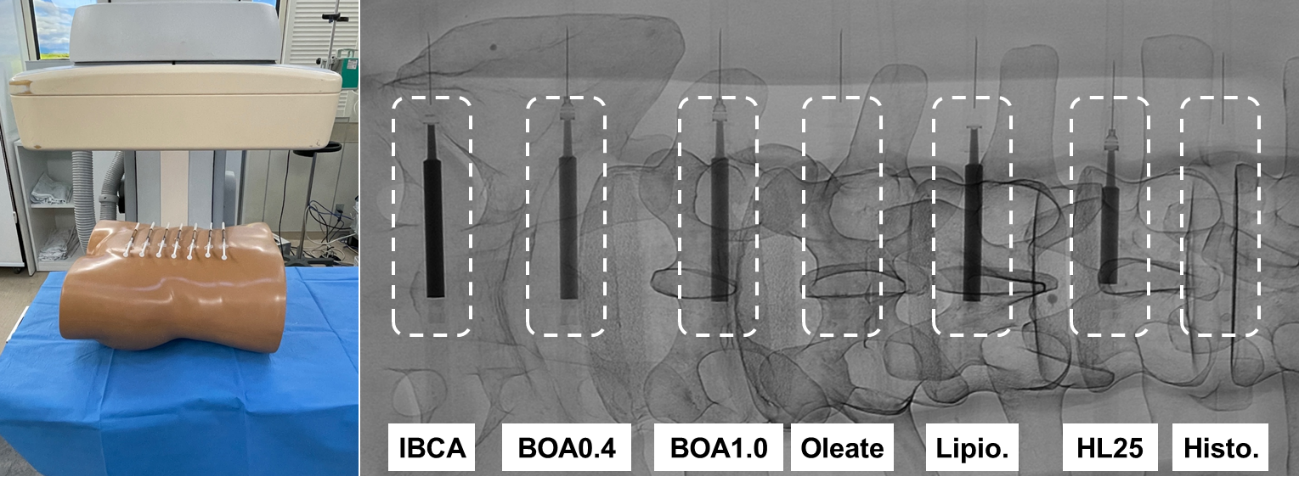
**

**Figure S5.** *In vitro* X-ray visibility evaluation of embolic compositions against soft tissue phantom using a real-time C-arm X-ray device.

**Figure S6.**


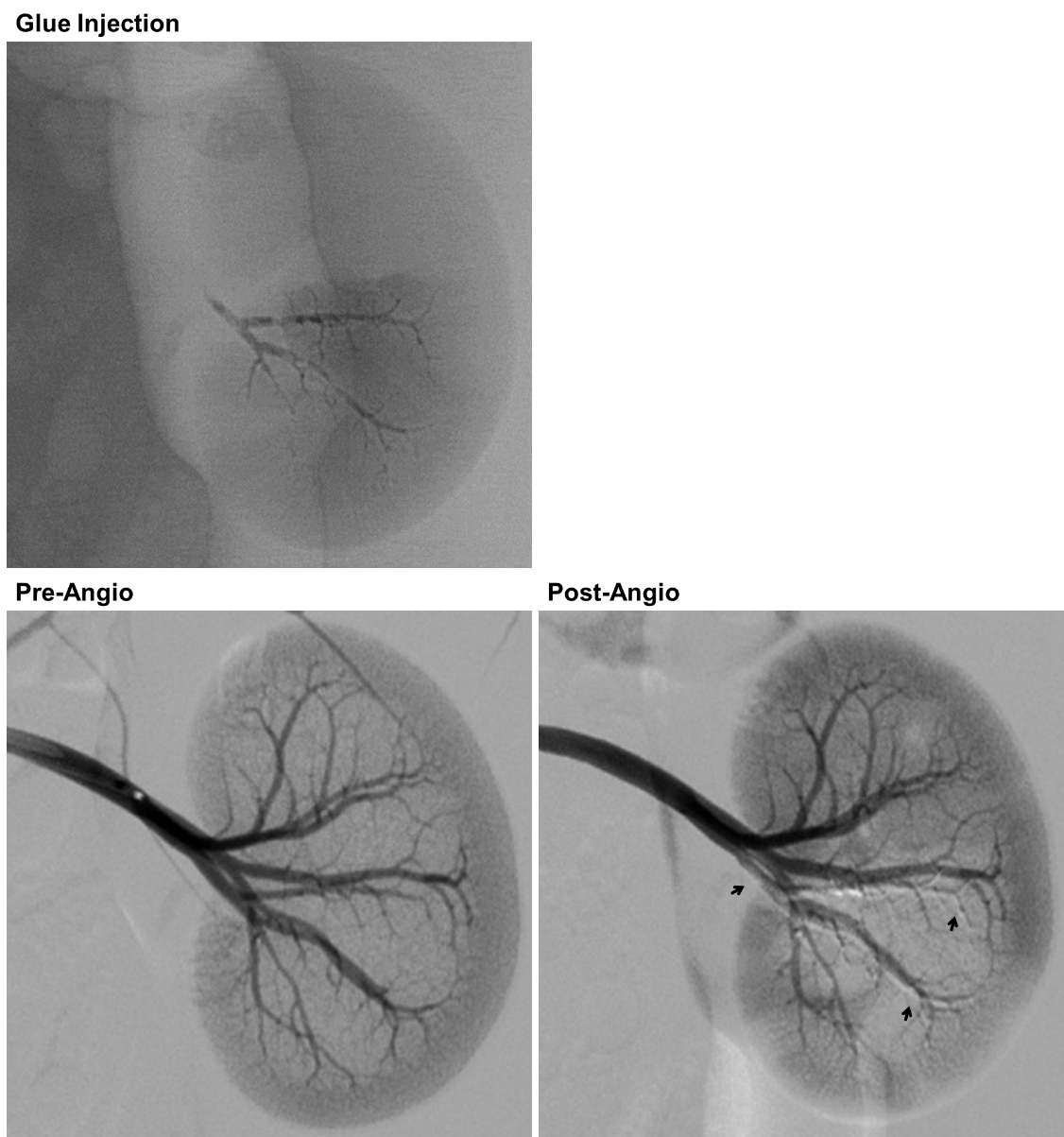


**Figure S6.** A real-time X-ray image of HL25-injected rabbit renal artery and DSA analysis before and after the embolization using HL25 (black arrow = embolized region).

**Figure S7.**


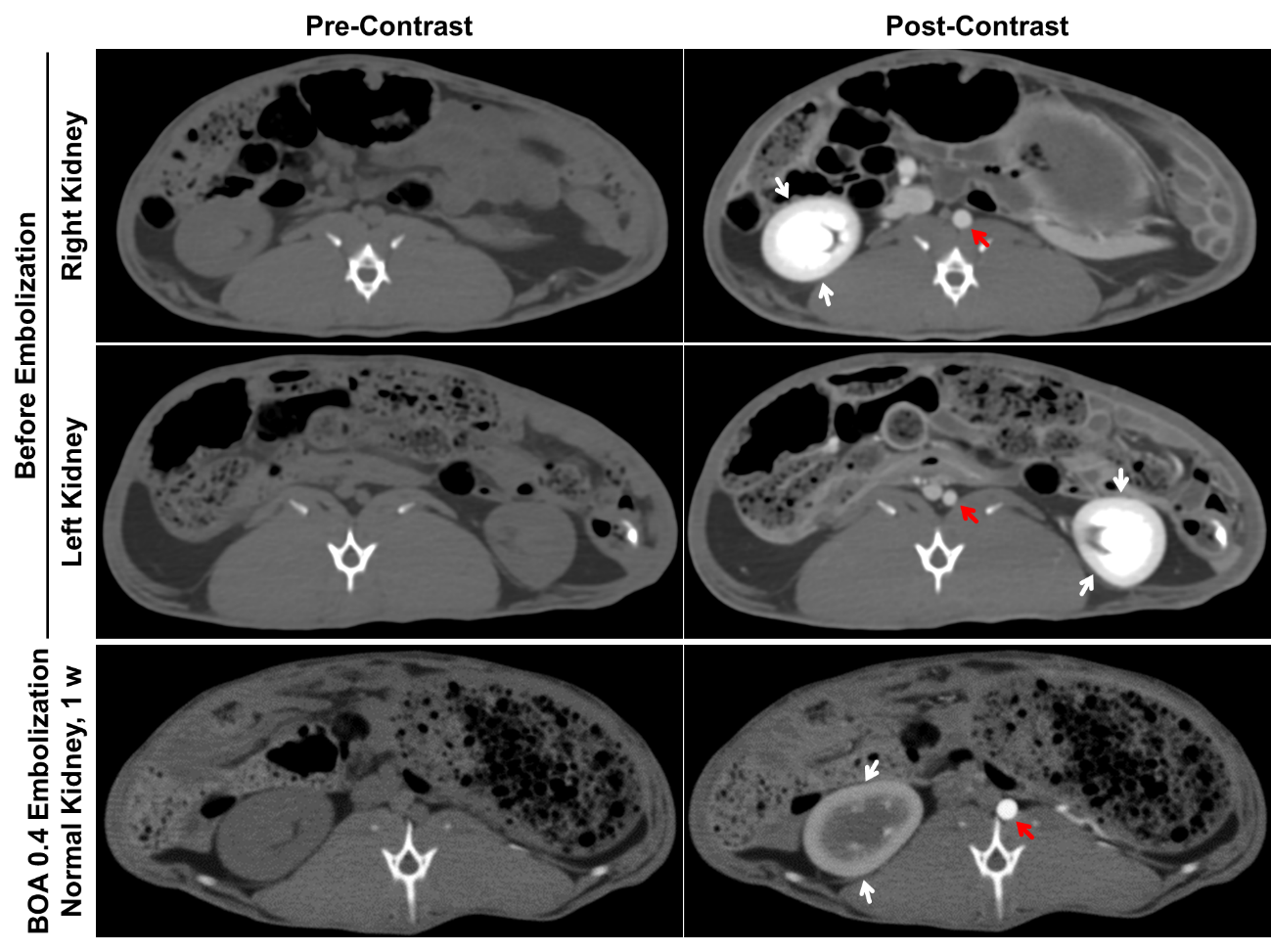


**Figure S7.** Abdominal CT images of the recipient rabbit before and 1 w after the embolization of the right kidney using BOA0.4, showing the contrast enhancement with Visipaque injection (red arrow = aorta, white arrow = non-embolized region).

**Figure S8.**


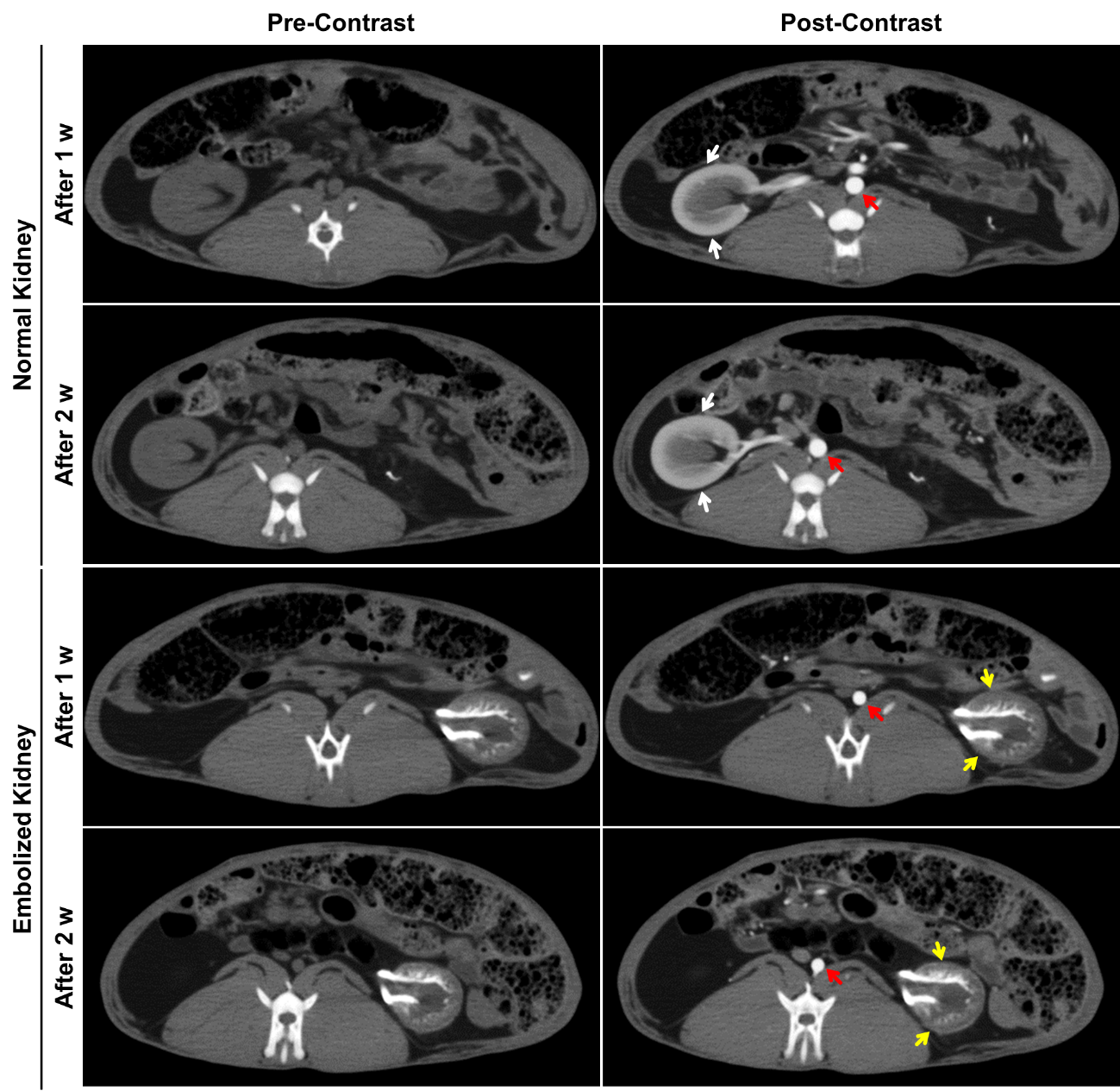


**Figure S8.** Abdominal CT images of the recipient rabbit 1 and 2 w post-embolization of the right kidney using BOA1.0, showing the contrast enhancement with Visipaque injection (red arrow = aorta, white arrow = non-embolized region, yellow arrow = embolized region).

**Figure S9.**


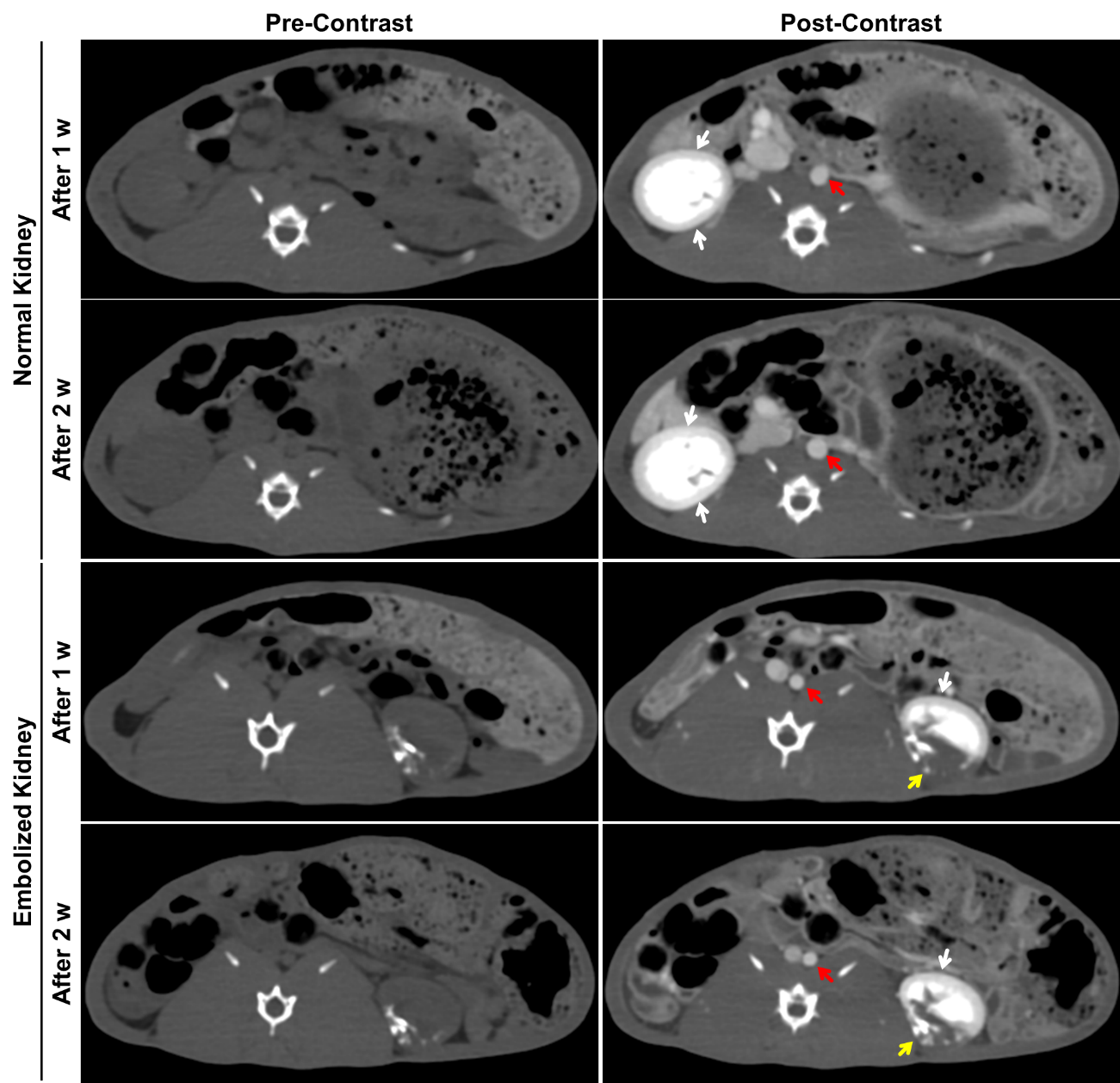


**Figure S9.** Abdominal CT images of the recipient rabbit at 1 and 2 w post-embolization of the right kidney using HL25, showing the contrast enhancement with Visipaque injection (red arrow = aorta, white arrow = non-embolized region, yellow arrow = embolized region).

**Figure S10.**


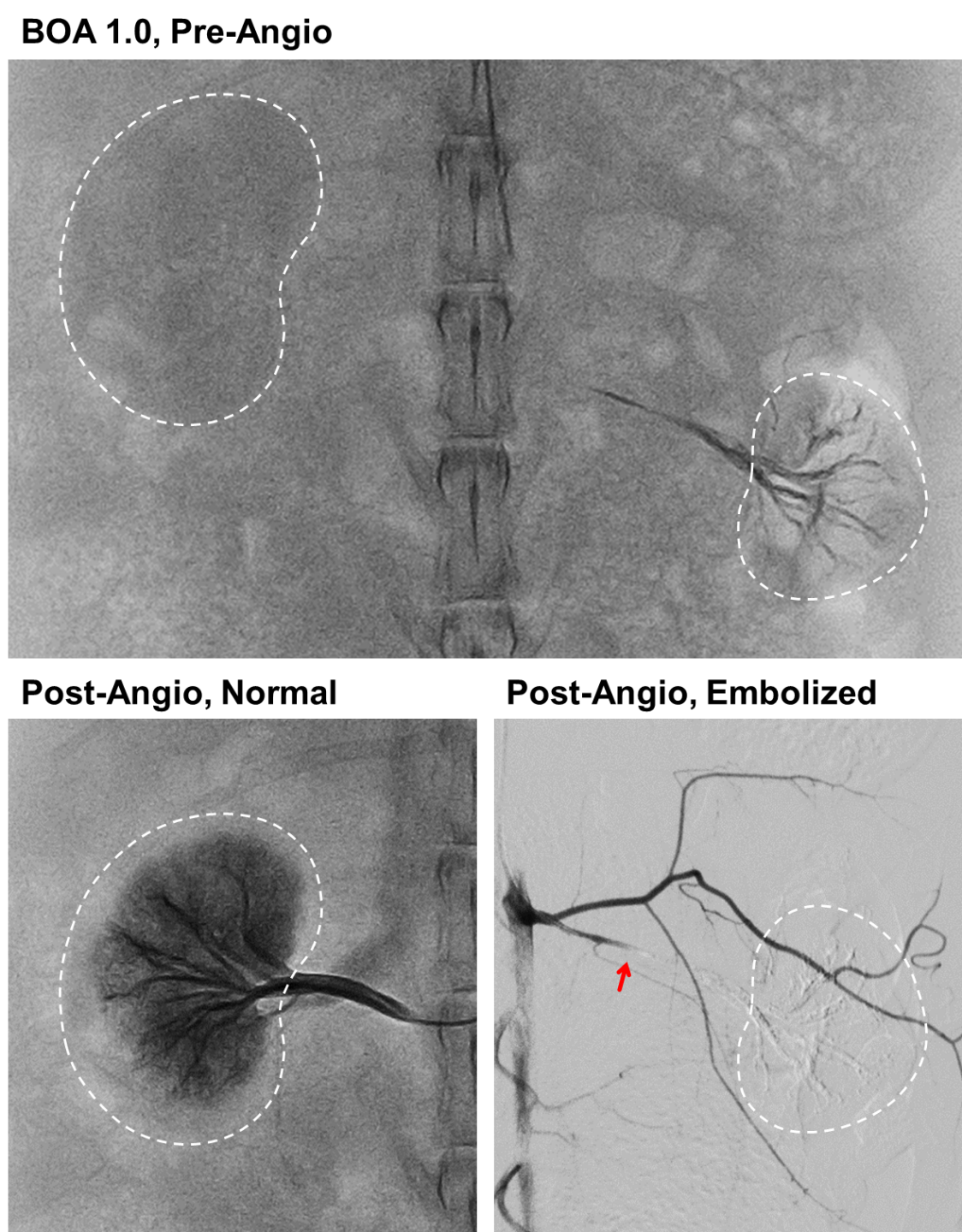


**Figure S10.** A real-time X-ray image of normal and embolized kidneys 4 w after the embolization with BOA1.0 and their DSA analysis (white dashed line = kidney, red arrow = bloodstream stagnation).

**Figure S11.**


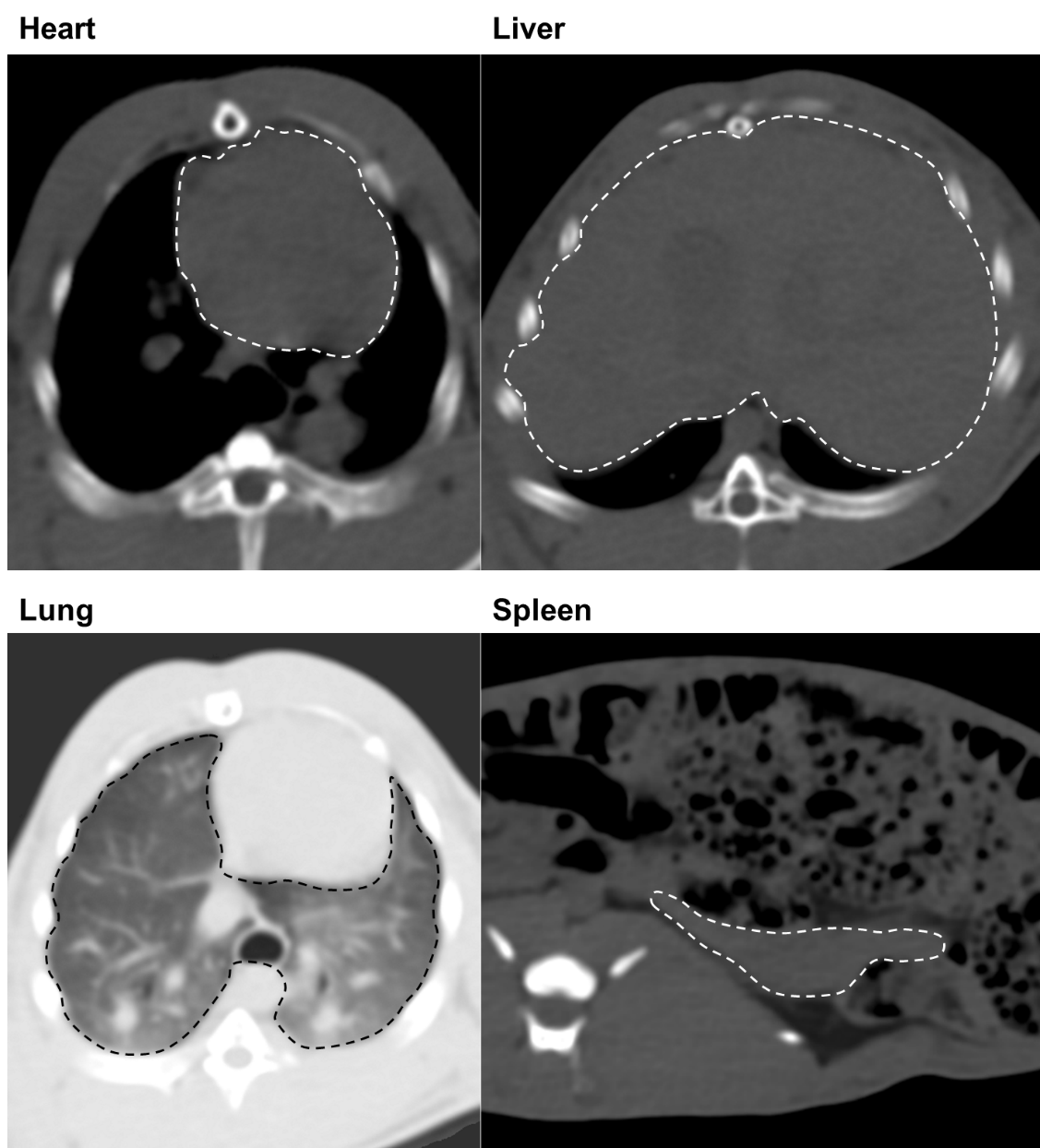


**Figure S11.** Abdominal CT images of recipient rabbit 2 w after the embolization with HL25, showing the absence of non-target embolization in main organs (dashed line = organ).

**Figure S12.**

**
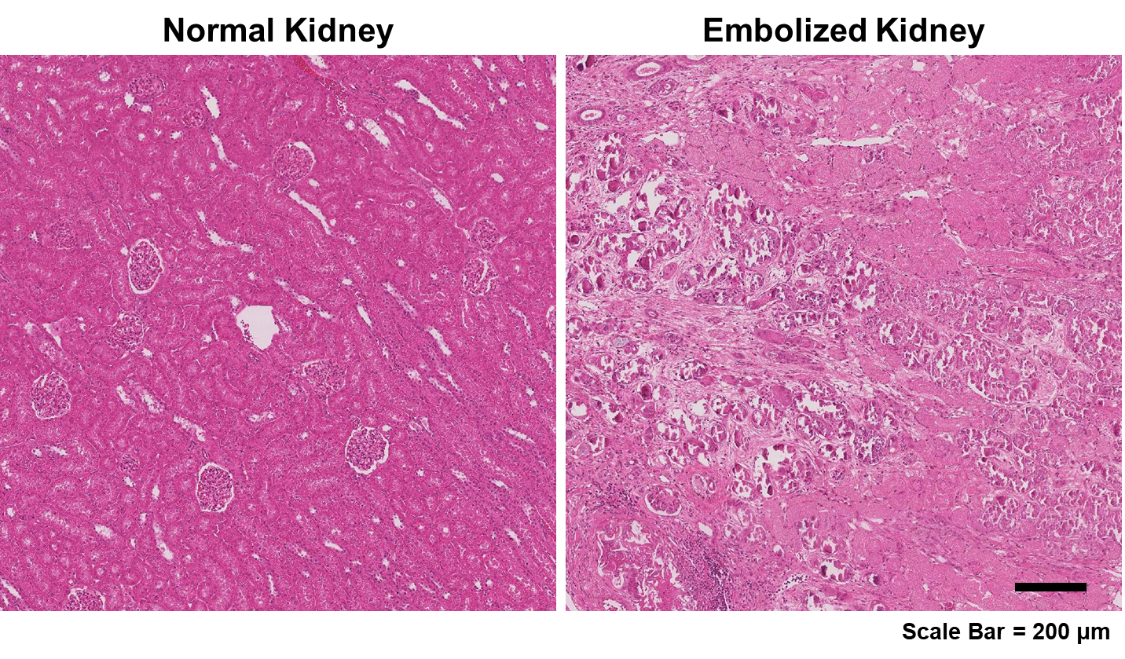
**

**Figure S12.** H&E histological analysis of normal and embolized kidneys extracted from rabbits with HL25 embolization after 4 w.

**Figure S13.**

**
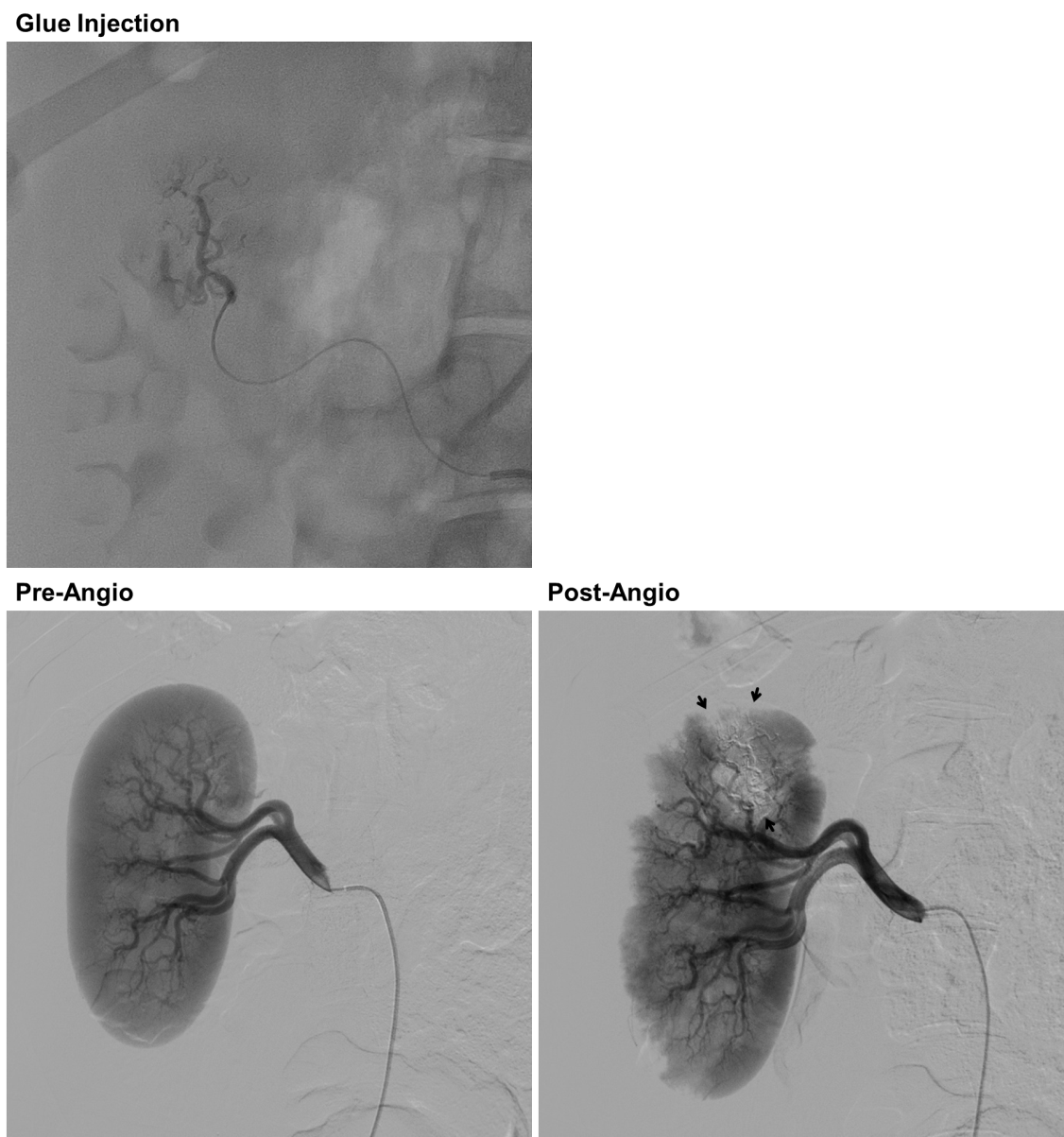
**

**Figure S13.** A real-time X-ray image of HL25-injected swine renal artery and DSA analysis before and after the embolization using HL25 (black arrow = embolized region).

**Figure S14.**

**
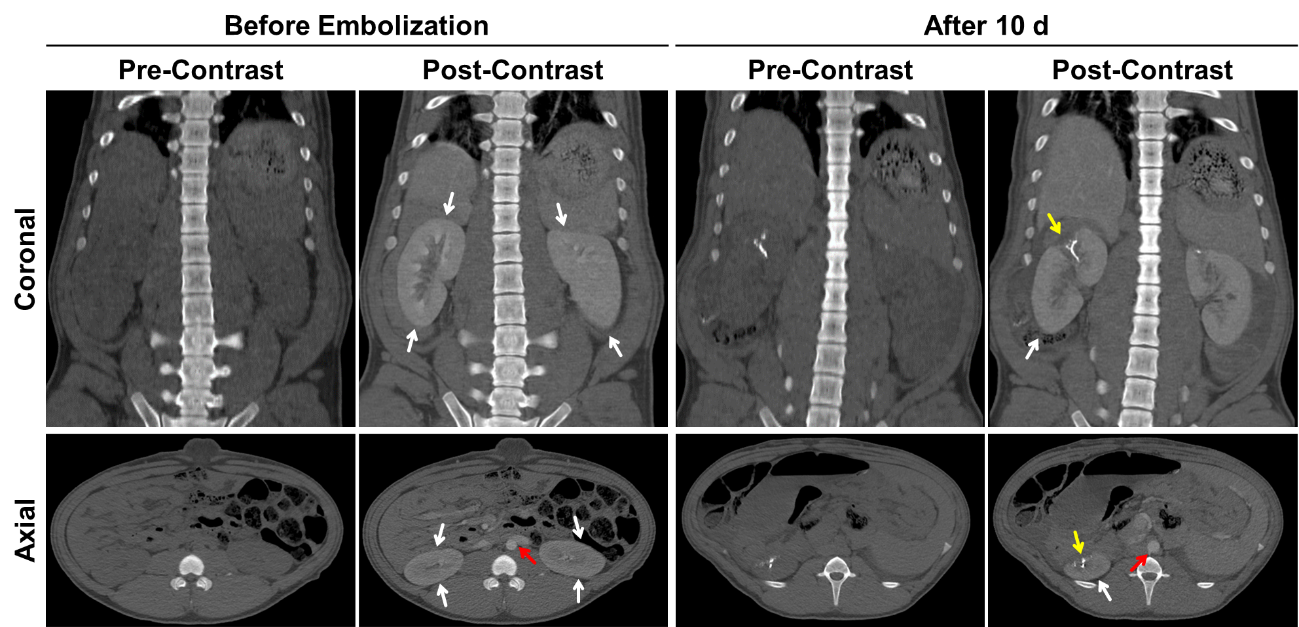
**

**Figure S14.** Abdominal CT images of the recipient swine before and 10 d after the embolization of the left kidney using HL25, showing the contrast enhancement with Visipaque injection (red arrow = aorta, white arrow = non-embolized region, yellow arrow = embolized region).

**Figure S15.**

**
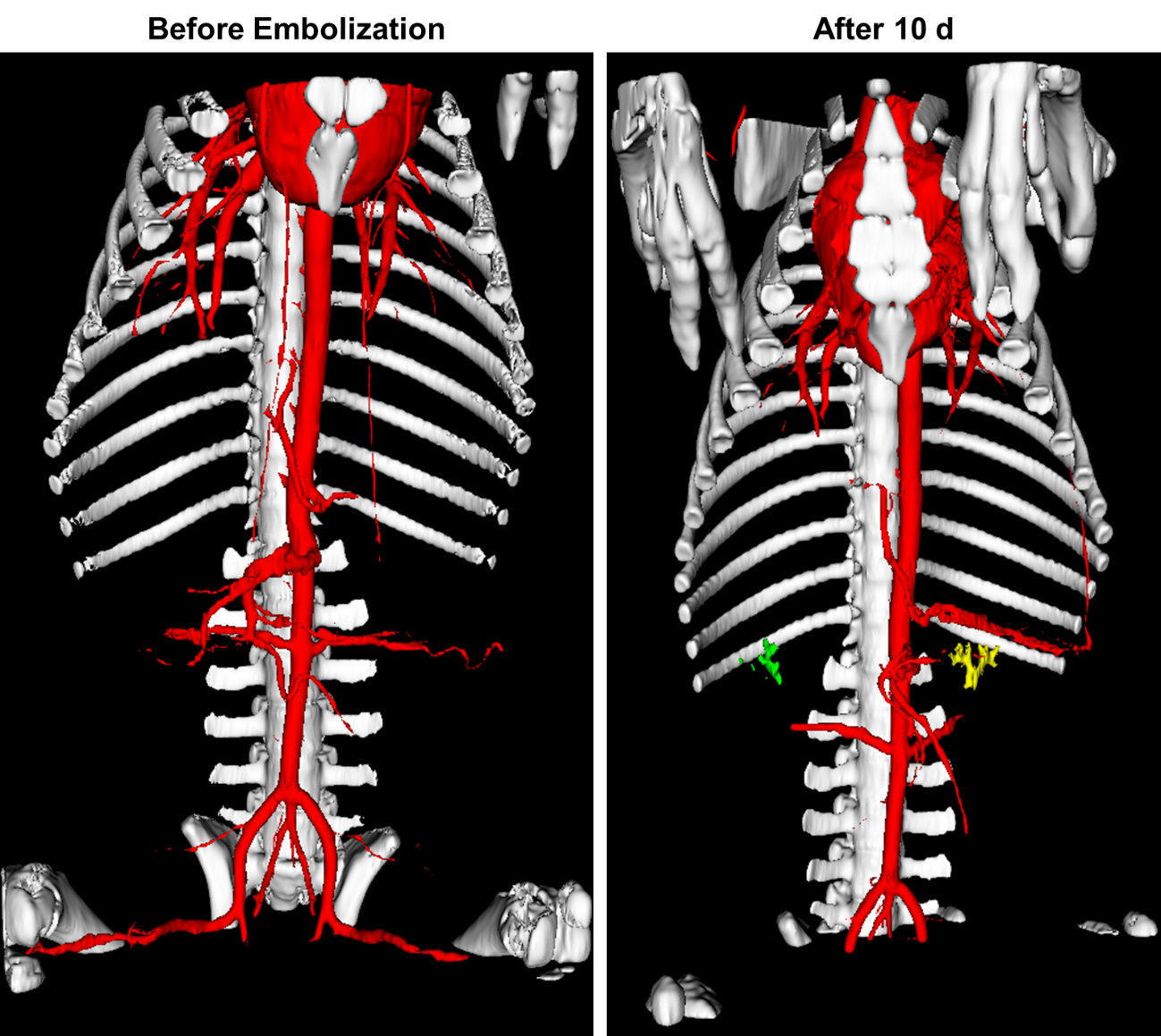
**

**Figure S15.** 3D-constructed CT images showing the location and morphology of HL25 (green) and BOA0.4 (yellow) embolic casts.

**Figure S16.**

**
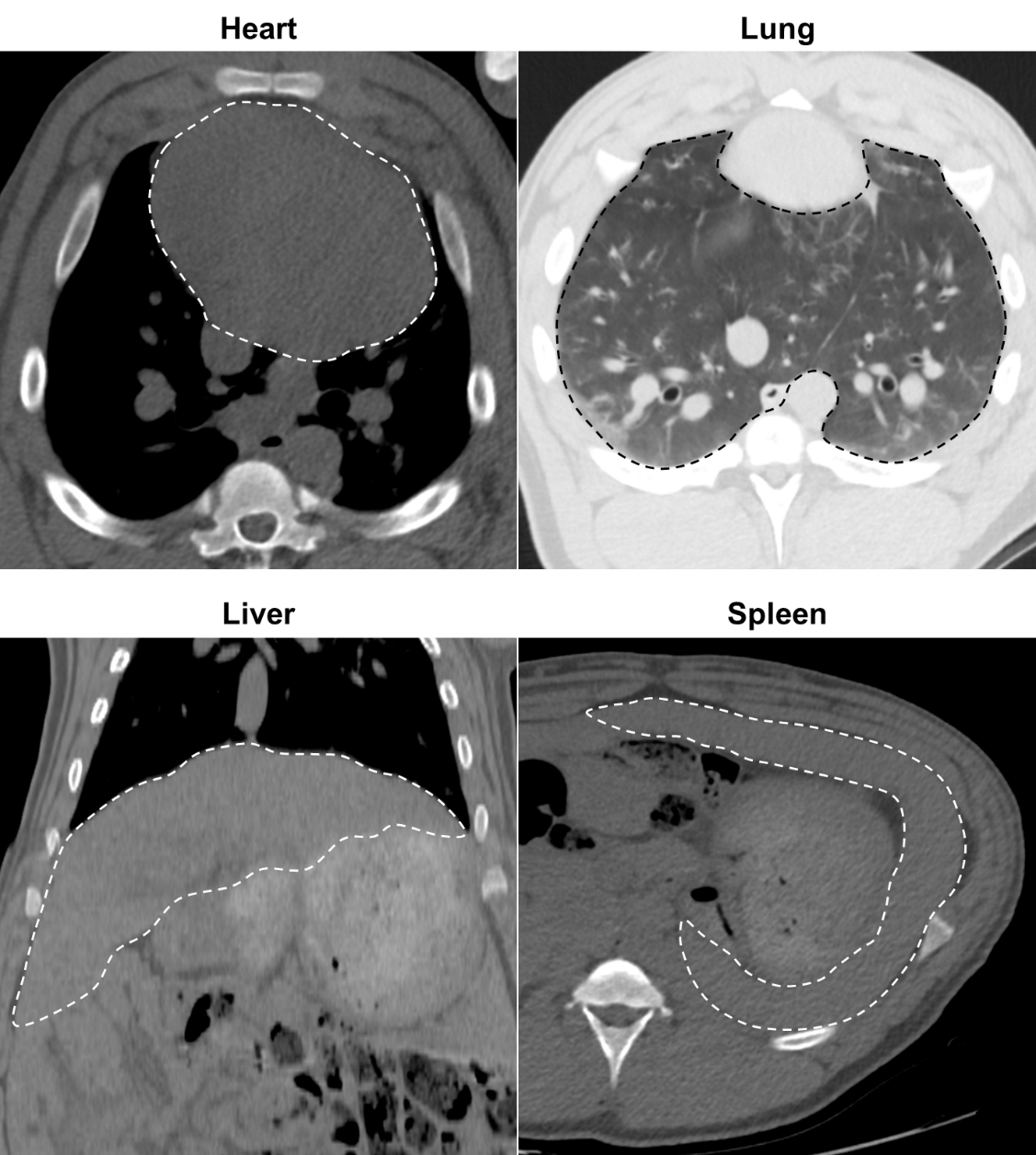
**

**Figure S16.** Abdominal CT images of recipient swine before the embolization (dashed line = organ).

**3. Captions for Supplementary videos**

**Video S1.** DSA analysis of rabbit renal artery before and after the embolization with BOA0.4.

**Video S2.** DSA analysis of rabbit renal artery before and after the embolization with BOA1.0.

**Video S3.** DSA analysis of normal (right kidney) and embolized (left kidney) rabbit renal arteries 4 w after the embolization with BOA0.4.

**Video S4.** DSA analysis of normal (right kidney) and embolized (left kidney) rabbit renal arteries 4 w after the embolization with BOA1.0.

**Video S5.** DSA analysis of swine renal artery before and after the embolization with BOA 1.0.
